# Mesalazine for People with Diverticular Disease: A Systematic Review of Randomized Controlled Trials

**DOI:** 10.1155/2018/5437135

**Published:** 2018-09-16

**Authors:** Andrea Iannone, Marinella Ruospo, Germaine Wong, Michele Barone, Mariabeatrice Principi, Alfredo Di Leo, Giovanni F. M. Strippoli

**Affiliations:** ^1^Section of Gastroenterology, Department of Emergency and Organ Transplantation, University of Bari, Italy; ^2^Diaverum Medical Scientific Office, Lund, Sweden; ^3^Department of Translational Medicine, Amedeo Avogadro University of Eastern Piedmont, Novara, Italy; ^4^Sydney School of Public Health, University of Sydney, Australia; ^5^Centre for Transplant and Renal Research, Westmead Hospital, Sydney, Australia; ^6^Diaverum Academy, Lund, Sweden; ^7^Section of Nephrology, Department of Emergency and Organ Transplantation, University of Bari, Italy

## Abstract

**Background:**

Diverticular disease treatment is limited to fibres, antibiotics, and surgery. There is conflicting evidence on mesalazine benefits and harms.

**Aim:**

We systematically reviewed current evidence on benefits and harms of mesalazine versus all other treatments in people with diverticular disease.

**Methods:**

We searched MEDLINE, EMBASE, CENTRAL, ClinicalTrials.gov for studies published to July 2018. We estimated risk ratios (RR) for dichotomous outcomes (disease remission/recurrence, acute diverticulitis in symptomatic uncomplicated diverticular disease, need for surgery/hospitalization, all-cause/disease-related mortality, adverse events), mean differences (MD) or standardized MD (SMD) for continuous outcomes (quality of life, symptoms score, time to recurrence/remission), and their 95% confidence intervals (CI) using random-effects models. We quantified heterogeneity by Chi^2^ and I^2^ tests. We performed subgroup analyses by disease subtype, comparator, follow-up duration, mesalazine dose, and mode of administration.

**Results:**

We identified 13 randomized trials (n=3028 participants). There was a higher likelihood of disease remission with mesalazine than controls in acute uncomplicated diverticulitis (1 trial, 81 participants, RR=2.67, 95%CI=1.05-6.79), but not in symptomatic uncomplicated diverticular disease (1 trial, 123 participants, RR=1.04, 95%CI=0.81-1.34). There was a lower likelihood of disease recurrence with mesalazine than controls in symptomatic uncomplicated diverticular disease (2 trials, 216 participants, RR=0.52, 95%CI=0.28-0.97), but not in acute uncomplicated diverticulitis (7 trials, 2196 participants, RR=0.90, 95%CI=0.61-1.33). There was no difference in the likelihood of developing acute diverticulitis in symptomatic uncomplicated diverticular disease between the two groups (3 trials, 484 participants, RR=0.26, 95%CI=0.06-1.20). There was a higher global symptoms score reduction with mesalazine than controls in symptomatic uncomplicated diverticular disease (2 trials, 326 participants, SMD=-1.01, 95%CI=-1.51,-0.52) and acute uncomplicated diverticulitis (2 trials, 153 participants, SMD=-0.56, 95%CI=-0.88,-0.24).

**Conclusions:**

Mesalazine may reduce recurrences in symptomatic uncomplicated diverticular disease. There is uncertainty on the effect of mesalazine in achieving diverticular disease remission. Mesalazine may not prevent acute diverticulitis in symptomatic uncomplicated diverticular disease.

## 1. Introduction

The prevalence of colonic diverticulosis increases with age, ranging from approximately 30% in people aged 50-59 years to around 70% in those older than 80 years [1]. Some 20% of people with diverticulosis experience symptoms related to diverticular mucosa inflammation, ranging from mild abdominal pain to severe complications [2]; this condition is defined diverticular disease [3]. Low dietary fibre intake, gut microbiota alteration, obesity and changes in colonic motility, and sensitivity play a role in the development of symptoms and complications in diverticular disease [4]. About 2% of people with diverticular disease require hospital admission, 0.5% need surgery, and 1% die during hospitalization [5, 6].

Current management of diverticular disease focuses on dietary and pharmacological interventions, such as rifaximin and systemic antibiotics. Some guidelines [4, 7, 8] suggest the combination of high-fibre diet and rifaximin in symptomatic uncomplicated diverticular disease for symptoms relief and acute diverticulitis prevention. In case of acute diverticulitis, differently from early recommendations [3, 9–11], antibiotics are currently suggested only if complications arise and surgical interventions are required [4, 7, 8, 12–16]. No treatment prevents diverticular disease recurrences or complications (Supplementary Table 1).

Given the existing uncertainties, mesalazine, an anti-inflammatory drug, has been purported as a promising intervention in diverticular disease. Studies with different designs have investigated the use of mesalazine in diverticular disease and found contradicting results on achieving disease remission and preventing recurrences. Despite these individual conflicting findings, some clinical practice guidelines now recommend mesalazine for symptom relief in symptomatic uncomplicated diverticular disease [7, 14] or recurrence prevention in acute uncomplicated diverticulitis [12].

We systematically reviewed the totality of evidence on the benefits and harms of mesalazine versus all other treatments in people with diverticular disease, to inform clinical decisions.

## 2. Materials and Methods

The review was conducted according to the Preferred Reporting Items for Systematic Reviews and Meta-Analyses (PRISMA) statement [17].

### 2.1. Data Sources and Searches

A comprehensive literature search for randomized controlled trials of mesalazine in diverticular disease was performed in MEDLINE (1946 to July 2018), EMBASE (1996 to July 2018), the Cochrane Central Register of Controlled Trials (CENTRAL, from inception to July 2018), and ClinicalTrials.gov (searched on July 2018) using highly sensitive search strategies designed by an information specialist (RM). Searches were performed without language restriction. Relevant studies were also searched from reference lists of identified trials and guidelines. Search strategies are outlined in Supplementary Table 2.

### 2.2. Study Selection

Two reviewers (AI, MR) independently screened the searches by title and abstract, then the full text, to identify potentially eligible trials. We included any randomized controlled trials which compared mesalazine, at any dose, mode, and duration of administration with any intervention for the treatment of people with diverticular disease. Diverticular disease was defined as the presence of any colonic diverticula-related symptoms [3]. Considering the lack of a standardized definition of symptomatic uncomplicated diverticular disease, we considered this subtype of diverticular disease as the occurrence of nonspecific diverticular disease-related abdominal symptoms, such as lower abdominal pain and altered bowel habit, in the absence of macroscopic colonic mucosa alterations [4, 7, 14]. Acute uncomplicated diverticulitis was defined as the presence of macroscopic diverticula inflammation which causes symptoms, such as fever, lower abdominal pain, and leukocytosis in people with diverticular disease [4, 8, 14, 15]. The development of complications, such as abscess, perforation, obstruction, or bleeding, characterized by acute complicated diverticulitis [2]. We included studies in which colonic diverticulosis diagnosis was performed by endoscopy and/or radiologic imaging.

We excluded trials not involving any arm treated with mesalazine alone or comparing this treatment only with a combination therapy including mesalazine. We also excluded trials investigating mesalazine in people with segmental colitis associated with diverticulosis, a chronic inflammatory process involving the interdiverticular mucosa, since it is considered a distinct pathologic entity [4, 18].

### 2.3. Data Extraction and Quality Assessment

Two reviewers (AI, MR) independently assessed each randomized controlled trial for eligibility and extracted data on study, population, intervention (experimental and comparator treatments), and outcomes characteristics.

#### 2.3.1. Outcomes Measures

We evaluated the number of participants achieving diverticular disease remission, developing diverticular disease recurrence, and experiencing acute diverticulitis in symptomatic uncomplicated diverticular disease. We considered disease remission as the disappearance of all diverticula-related symptoms after the beginning of treatment with mesalazine or other interventions; relapse of diverticula-related symptoms in asymptomatic participants, after the beginning of the treatment with mesalazine or other interventions, was defined as disease recurrence.

Other outcomes were quality of life and symptoms relief assessed by any instrument reported in the trials, the number of participants requiring surgery and needing hospitalization, all-cause and diverticular disease-related mortality, any adverse events and time to diverticular disease recurrence, diverticular disease remission, and surgery and acute diverticulitis onset in symptomatic uncomplicated diverticular disease. We extracted outcome data according to the maximum period of observation in each trial.

If published outcome data were not reported or provided in sufficient detail, an author (AI) contacted trial investigators one time by electronic mail requesting any relevant additional information. When obtained, information was included in the analyses.

#### 2.3.2. Risk of Bias Assessment

We assessed the study level risk of bias with the Cochrane risk of bias tool, including the domains of random sequence generation, allocation concealment, blinding of participants or investigators, blinding of outcome assessment, completeness of outcome data, selective reporting, and other threats to validity.

Review authors resolved any disagreement in data extraction and quality assessment through discussion and involvement of an arbitrator (GFMS).

### 2.4. Data Synthesis and Analysis

We estimated risk ratios (RR) for dichotomous outcomes and mean differences (MD) for continuous outcomes, with their 95% confidence intervals (95%CI), in individual studies. If continuous outcomes were measured using different scales across individual studies, we calculated standardized mean differences (SMD) with 95%CI. We assessed pooled estimates using the DerSimonian and Laird random-effects model [19].

We formally estimated heterogeneity of intervention effects among studies with the Chi^2^ (Cochran Q) and the I^2^ statistics.

We performed prespecified subgroup analyses by subtype of diverticular disease (symptomatic uncomplicated diverticular disease, acute uncomplicated diverticulitis or acute complicated diverticulitis), type of comparator (probiotics, rifaximin, systemic antibiotics, placebo, or no treatment), follow-up duration (less than 3 months, 3 to 6 months, 6 to 12 months, or more than 12 months), mesalazine dose (800 to 1600 mg, more than 1600 to 2400 mg, or more than 2400 mg), and mode of administration (continuous or cyclic). All subgroup analyses were stratified by subtype of diverticular disease.

To avoid double-counting of participants in trials comparing mesalazine with more than one intervention, we divided the events and total number of participants allocated to the mesalazine arm according to the number of times that data from this group was used in each analysis. We used the same method for trials including more than one mesalazine arm, with different drug doses, dividing the events and total number of patients allocated to the comparator arm according to the number of times that data from this group was used in each analysis. We estimated differences among subgroups by the Mantel-Haenszel test. We planned to explore publication bias with funnel plots where at least 10 studies were included [20].

We rated quality of evidence according to the Grades of Recommendation, Assessment, Development and Evaluation (GRADE) approach [21].

All analyses were performed using RevMan 5.3 (The Cochrane Collaboration, Copenhagen, Denmark).

## 3. Results

### 3.1. Search Results

**Figure 1:**
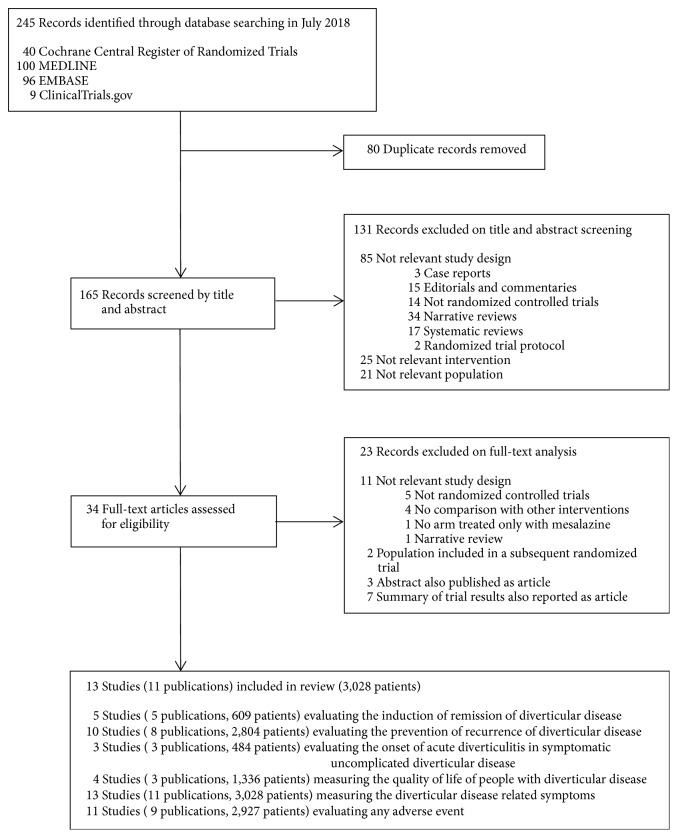
Flow diagram of search results and selection of included studies.

Of 245 articles identified electronically, 80 were duplicates and 131 were ineligible after abstract review (Figure 1). The remaining 34 articles were retrieved and reviewed in full text form, with 13 trials (11 publications) [22–32], enrolling a total of 3028 participants with diverticular disease, included. We received additional unpublished information from the authors of 3 trials [24, 25, 29]. Two trials published in 2007 by the same group [24, 25] had only 8 participants included in both studies; thus they were considered as separate trials.

### 3.2. Characteristics of Included Studies

Table 1 shows the characteristics of populations, intervention, and comparators in the included studies. Sample size in the trials varied from 43 to 592. Six trials (46%) [23–27, 30] enrolled people with symptomatic uncomplicated diverticular disease and 7 (54%) [22, 28, 29, 31, 32] enrolled people with acute uncomplicated diverticulitis. No study included patients with acute complicated diverticulitis. Mesalazine was compared with probiotics in 1 (8%) [23], rifaximin in 2 (15%) [24, 25], placebo in 8 (61%) [26–29, 31, 32], no treatment in 1 (8%) [22], and both probiotics and placebo in 1 (8%) trials [30]. No study compared mesalazine with systemic antibiotics.

### 3.3. Quality of Studies

The risk of bias of the 13 included trials is summarized in Supplementary Figure 1.

Random sequence generation was adequate in 7 (54%) trials and unclear in 6 (46%). Allocation concealment was adequate in 2 (15%) studies and unclear in 11 (85%). Blinding of participants and investigators was adequate in 8 (62%) trials, inadequate in 2 (15%), and unclear in the remaining 3 (23%). Seven (54%) trials had adequate blinding of outcome assessment, while in 6 (46%) this was unclear. Analysis was by intention-to-treat in 5 (38%) and both intention-to-treat and per protocol in 6 (46%) trials, while this was unclear in the remaining 2 (16%). Withdrawal of participants from analyses was <10% in 7 (54%) trials and >10% in 6 (46%).

Four (31%) out of 13 included trials were at low risk of bias for most of the quality domains [27, 30–32].

### 3.4. Outcomes

Table 2 summarizes key results of the comparison of mesalazine versus control interventions for diverticular disease, by disease subtype, and the grading of evidence assessment, according to the GRADE approach. Complete results of the comparison of mesalazine versus control interventions for diverticular disease are shown in Supplementary Table 3.

#### 3.4.1. Disease Remission

There was no statistically significant difference in the likelihood of achieving diverticular disease remission between mesalazine and control interventions (2 trials, 204 participants, RR=1.51, 95%CI=0.57-3.98, and I^2^=76%).

#### 3.4.2. Disease Recurrence

**Figure 2:**
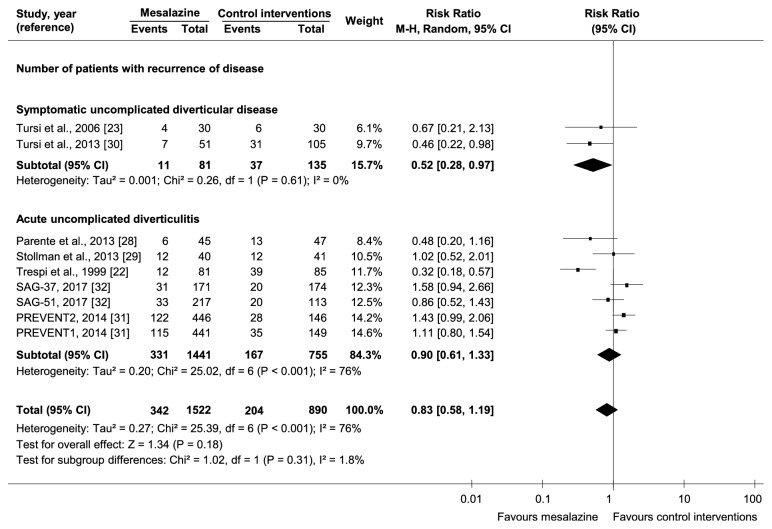
Comparative effectiveness of mesalazine versus control interventions by subtype of diverticular disease on the number of participants developing disease recurrence.

There was no statistically significant difference in the likelihood of diverticular disease recurrence between mesalazine and control interventions (9 trials, 2414 participants, RR=0.83, 95%CI=0.58-1.19, and I^2^=73%) (Figure 2). One trial [22] had participants treated only for 2 months and then followed up for 48 months, leading to uncertainty on the association between mesalazine and prevention of disease recurrence. Another trial [28] enrolled only people at their first diagnosis of acute uncomplicated diverticulitis, a condition with an increased response to mesalazine [2].

#### 3.4.3. Acute Diverticulitis Onset in Symptomatic Uncomplicated Diverticular Disease

There was no statistically significant difference in the likelihood of developing acute diverticulitis in symptomatic uncomplicated diverticular disease between mesalazine and control interventions (3 trials, 484 participants, RR=0.26, 95%CI=0.06-1.20, and I^2^=0%).

#### 3.4.4. Quality of Life

Four trials [24, 28, 31] compared mesalazine with control interventions on quality of life (Table 3). One trial [24] included participants with symptomatic uncomplicated diverticular disease, while 3 [28, 31] included people with acute uncomplicated diverticulitis. There was a higher improvement of physical functioning (p<0.05) and general health (p=0.01) in the mesalazine than rifaximin group at 6 months in people with symptomatic uncomplicated diverticular disease [24]. Conversely, there was no significant difference in quality of life scores between the two groups in acute uncomplicated diverticulitis [28, 31].

#### 3.4.5. Symptoms Relief

**Figure 3:**
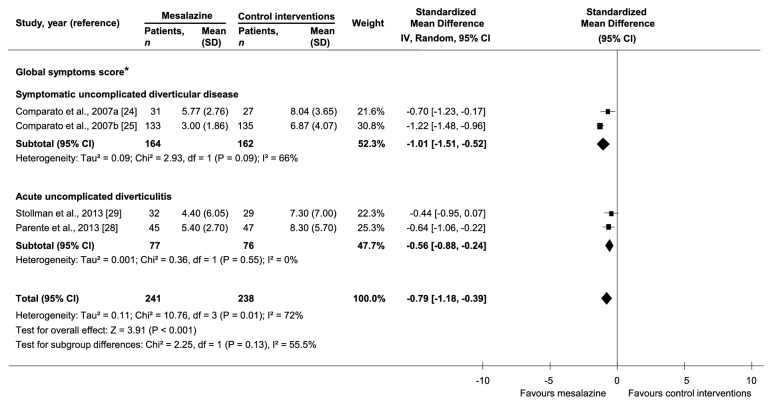
Comparative effectiveness of mesalazine versus control interventions by subtype of diverticular disease on global symptoms score. *∗*There was not statistically significant difference in baseline global symptoms score between mesalazine and control interventions arms in all included studies.

Thirteen trials [22–32] compared mesalazine with control interventions on symptoms relief (Supplementary Table 4). There was a statistically significant reduction of diverticula-related symptoms with mesalazine compared to control interventions in 4 out of 6 trials on symptomatic uncomplicated diverticular disease and in 2 out of 7 trials on acute uncomplicated diverticulitis. In the analysis of the 4 [24, 25, 28, 29] trials reporting a global symptoms score, there was a lower mean score at maximum follow-up with mesalazine than control interventions (4 trials, 479 participants, SMD=-0.79, 95%CI=-1.18,-0.39, and I^2^=72%) (Figure 3). Baseline global symptoms score was not statistically different between mesalazine and control interventions arms in all included studies.

A summary of trial results for other outcomes is reported in Supplementary Table 3.

#### 3.4.6. Subgroup Analyses

Subgroup analyses by subtype of diverticular disease, type of control intervention, follow-up duration, mesalazine dose, and mode of administration are reported in Supplementary Tables 3, 5, 6, 7, and 8, respectively.

*Subtype of Diverticular Disease*. There was a higher likelihood of achieving remission with mesalazine than control interventions in acute uncomplicated diverticulitis (1 trial, 81 participants, RR=2.67, and 95%CI=1.05-6.79), but not in symptomatic uncomplicated diverticular disease (1 trial, 123 participants, RR=1.04, and 95%CI=0.81-1.34). There was no significant interaction between subgroups (p=0.06). There was a lower likelihood of recurrence with mesalazine than control interventions in symptomatic uncomplicated diverticular disease (2 trials, 216 participants, RR=0.52, and 95%CI=0.28-0.97), but not in acute uncomplicated diverticulitis (7 trials, 2196 participants, RR=0.90, and 95%CI=0.61-1.33) (Figure 2). There was no significant interaction between subgroups (p=0.14). There was a lower mean score at maximum follow-up with mesalazine than control interventions in both symptomatic uncomplicated diverticular disease (2 trials, 326 participants, SMD=-1.01, and 95%CI=-1.51,-0.52) and acute uncomplicated diverticulitis (2 trials, 153 participants, SMD=-0.56, and 95%CI=-0.88,-0.24) (Figure 3). There was no significant interaction between subgroups (p=0.13). 

*Type of Comparator*. There was a lower likelihood of symptomatic uncomplicated diverticular disease recurrence with mesalazine only in the comparison with placebo (1 trial, 101 participants, RR=0.33, and 95%CI=0.13-0.86). There was no significant interaction among subgroups (p=0.22). In acute uncomplicated diverticulitis, there was a lower likelihood of recurrence with mesalazine only in the comparison with no treatment (1 trial, 166 participants, RR=0.32, and 95%CI=0.18-0.57), with a statistically significant difference among subgroups (p=0.001). However, in the single study [22] comparing mesalazine with no treatment participants were treated only for 2 months and then followed up for 48 months, leading to uncertainty on the association between mesalazine and prevention of disease recurrence. 

*Follow-Up Duration*. In acute uncomplicated diverticulitis, there was a lower mean global symptoms score with mesalazine than control interventions only at long-term (more than 12 months) follow-up (1 trial, 92 participants, SMD=-0.64, and 95%CI=-1.06,-0.22). There was no significant interaction among subgroups (p=0.55). 

*Mode of Mesalazine Administration*. There was a lower likelihood of symptomatic uncomplicated diverticular disease recurrence with mesalazine than control interventions only with cyclic administration (1 trial, 156 participants, RR=0.46, and 95%CI=0.22-0.98). There was no significant interaction among subgroups (p=0.61).

Publication bias could not be assessed due to the paucity of data.

## 4. Discussion

We found that mesalazine may decrease recurrences in symptomatic uncomplicated diverticular disease, but not in acute uncomplicated diverticulitis. Subgroup analyses confirmed an effect in symptomatic uncomplicated diverticular disease only when mesalazine was compared with placebo or cyclically administrated. Based on current evidence from randomized trials, there is uncertainty on the effect of mesalazine in achieving remission in both symptomatic uncomplicated diverticular disease and acute uncomplicated diverticulitis. The development of acute diverticulitis in people with symptomatic uncomplicated diverticular disease may not be reduced by mesalazine. Mesalazine may improve quality of life in symptomatic uncomplicated diverticular disease and the global symptoms score in both subtypes of diverticular disease, with a larger effect in symptomatic uncomplicated diverticular disease.

In acute uncomplicated diverticulitis, existing systematic reviews either suggest a role for mesalazine in symptoms relief [33, 34] and recurrence prevention [33–36] or find no evidence supporting mesalazine use in the prevention of disease relapse [37–39]. In symptomatic uncomplicated diverticular disease, three systematic reviews found that mesalazine has a role for symptoms relief [34, 36, 40], recurrence prevention [34, 36], and prevention of acute diverticulitis onset [36, 40].

Most of these analyses primarily described single study results without performing quantitative syntheses of data [33–36, 40]. In two of these reviews evidence is derived from both randomized trials and observational studies [33, 36], which are an inadequate study design to address intervention questions. Existing reviews also focused on one diverticular disease subtype only [33, 35, 37–40], with methodological limitations in search strategies and study selection or in outcomes analysis, considering selected outcomes rather than the full benefits-harms trade-off for mesalazine [33–40].

We found that mesalazine may not reduce acute uncomplicated diverticulitis recurrence but may possibly lead to its remission. This last finding is based only on one trial [29] enrolling a small sample of people with 1-3 previous episodes of acute diverticulitis, a condition with a high likelihood of response to mesalazine due to the low-grade intestinal fibrosis [41]. Thus, the certainty of evidence for this outcome was graded as “very low” and we could not provide any suggestions/recommendations on the use of mesalazine in the acute phase of acute uncomplicated diverticulitis. Our analysis confirms the role of mesalazine in symptomatic uncomplicated diverticular disease for the prevention of recurrences, but not for acute diverticulitis onset. We also found that mesalazine may produce a higher reduction in patients' symptoms compared to control interventions in both disease subtypes, with a large difference in symptomatic uncomplicated diverticular disease and a medium difference in acute uncomplicated diverticulitis.

Benchmarked with recommendations from key international guideline agencies, our findings support the more recently guidelines which do not recommend mesalazine for the prevention of acute uncomplicated diverticulitis recurrence [4, 7, 14, 15] and acute diverticulitis onset in symptomatic uncomplicated diverticular disease [4]. Guideline recommendations on mesalazine to achieve symptomatic uncomplicated diverticular disease remission are conflicting [4, 7, 14]. We found no benefit of mesalazine in this setting, although uncertainty persists since only one trial with a short-term follow-up assessed this outcome [27]. Finally, the Italian Group on Diverticular Diseases [4] states that there is no clear evidence on mesalazine use to achieve acute uncomplicated diverticulitis remission and prevent symptomatic uncomplicated diverticular disease recurrence. We show that mesalazine may decrease recurrences in symptomatic uncomplicated diverticular disease, while there is uncertainty on its effect in achieving acute uncomplicated diverticulitis remission.

Based on the analysis of 13 randomized trials, we can suggest the use of mesalazine for prevention of symptomatic uncomplicated diverticular disease relapse, a condition with no effective therapeutic alternatives, although results from long-term high-quality randomized trials and cost-effectiveness analyses are needed before extending the indications of this drug.

To the best of our knowledge, this is the first systematic review which compares mesalazine with other interventions for all diverticular disease subtypes, with a comprehensive evaluation of benefits and harms of this treatment and a quantitative synthesis of data. We evaluated the totality of evidence, including 13 trials versus 2-8 in existing systematic reviews [33–40], and only randomized trials compared to mixed use of both cohort and randomized designs in some previous reviews.

Our study has some limitations. Most of assessed outcomes had low/very low certainty of evidence due to the small number of included trials at low risk of bias (4 out of 13) and their heterogeneity. One trial [32] was stopped due to futility before the 24-month planned follow-up, leading to possible underestimation of events in mesalazine and control interventions arms. Moderate/high heterogeneity was found in some outcome analyses, which was mainly due to the different disease subtypes of primary studies populations. Anticipating this potential source of heterogeneity, we preplanned to stratify all outcome and subgroup analyses by diverticular disease subtype. The criteria for the diagnosis of symptomatic uncomplicated diverticular disease varied across included studies, since the definition of this condition is not yet standardized. The diagnosis of diverticular disease was not standardized across included trials (use of endoscopy, radiologic imaging, or both). We could not analyze the impact of mesalazine formulations on the considered outcomes. Most included studies used pH-dependent preparations, with a similar drug release in the colon, while only two trials [31] used the multimatrix (MMX) mesalazine formulation. We could not evaluate the effect of mesalazine by diverticula localization. More than 90% of included patients in each trial had diverticula in the left colon and results based on disease localization were not provided. We could not evaluate some relevant outcomes (times to remission, acute diverticulitis development in symptomatic uncomplicated diverticular disease, surgery, and hospitalization), as these were not reported in included trials. Finally, we could not analyze the cost-effectiveness of the suggested therapeutic approach with mesalazine for the prevention of symptomatic uncomplicated diverticular disease recurrences because no trial provided data on costs.

There remains a need for high-quality randomized trials to further investigate the role of mesalazine in diverticular disease and, especially, its effects on achieving disease remission. These studies should also better clarify the impact of mesalazine on diverticular disease-related symptoms, quality of life, and costs in symptomatic uncomplicated diverticular disease. The role of mesalazine in acute complicated diverticulitis and the comparison between its cyclic versus continuous administration require further consideration. Finally, randomized trials including populations with right-sided disease, a condition with a described different pathogenesis from the left-sided disease, are warranted to evaluate any differences in benefits and harms of mesalazine.

## 5. Conclusions

Based on the totality of evidence, mesalazine may decrease symptomatic uncomplicated diverticular disease recurrence, whereas it may not prevent acute uncomplicated diverticulitis relapse. There is uncertainty on the effect of mesalazine in achieving remission in symptomatic uncomplicated diverticular disease and acute uncomplicated diverticulitis. The development of acute diverticulitis in people with symptomatic uncomplicated diverticular disease may not be reduced by mesalazine. Mesalazine may improve quality of life in symptomatic uncomplicated diverticular disease and patients' symptoms in both subtypes of diverticular disease, with a larger effect in symptomatic uncomplicated diverticular disease.

## Figures and Tables

**Table 1:** Characteristics of the randomized controlled trials comparing mesalazine with control interventions for diverticular disease.

**Study (reference)**	**Year**	**Number of patients**	**Mean age (SD)**	**Procedure used for diagnosis of diverticular disease**		**Characteristics of ** **diverticular disease**		**First diagnosis of diverticular disease (**%**)**	**Mean time from last episode of diverticular disease (SD), *days***	**Characteristics of experimental treatment (mesalazine)**		**Characteristics of control interventions**			**Treatment duration, *months***	**Follow-up duration,* months***
				**Endoscopy**	**Radiologic imaging**	**Subtype**	**Activity**			**Daily dose, *mg***	**Mode of administration**	**Type**	**Daily dose**	**Mode of administration**		
Trespi et al. [22]	1999	166	61.4 (6.4)	√	√	Acute uncomplicated diverticulitis	Remission	21.0	na	800	Continuous	No treatment	/	/	2	48
Tursi et al. [23]	2006	60^a^	67.5	√		Symptomatic uncomplicated diverticular disease	Remission	31.1	na	1600	Continuous	Lactobacillus casei sub-species DG	16 billion viable lyophilized bacteria	Cyclic (15 days/month)	12	12
Comparato et al. *a* [24]	2007	58^b^	67.3 (10.2)	√	√	Symptomatic uncomplicated diverticular disease	Active	na	na	800 or 1600	Cyclic (10 days/month)	Rifaximin	400 or 800 mg	Cyclic (10 days/month)	6	6
Comparato et al. *b* [25]	2007	268^b^	65.0 (7.2)–67.4 (9.1)	√	√	Symptomatic uncomplicated diverticular disease	Active	na	na	800 or 1600	Cyclic (10 days/month)	Rifaximin	400 or 800 mg	Cyclic (10 days/month)	12	12
Smith et al. [26]	2012	43	na	√		Symptomatic uncomplicated diverticular disease	Active	na	na	3000	Continuous	Placebo	/	Continuous	3	3
Kruis et al. [27]	2013	123	63.0 (8.6)- 62.0 (8.6)	√		Symptomatic uncomplicated diverticular disease	Active	na	na	3000	Continuous	Placebo	/	Continuous	6 weeks	6 weeks
Parente et al. [28]	2013	92^c^	61.5 (11.1)	√	√	Acute uncomplicated diverticulitis	Remission	100.0	<12 months	1600	Cyclic (10 days/month)	Placebo	/	Cyclic (10 days/month)	24	24
Stollman et al. [29]	2013	81^d^	56.1 (11.1)- 57.7 (12.8)		√	Acute uncomplicated diverticulitis	Active	49.6	na	2400	Continuous	Placebo	/	Continuous	3	12
Tursi et al. [30]	2013	156^e^	60-64^f^	√		Symptomatic uncomplicated diverticular disease	Remission	54.3	na	1600	Cyclic (10 days/month)	Placebo Lactobacillus casei sub-species DG	/ 24 billion viable lyophilized bacteria	Cyclic (10 days/month) Cyclic (10 days/month)	12	12
PREVENT1 [31]^g^	2014	590	55.3 (11.4)	√	√	Acute uncomplicated diverticulitis	Remission	58.1	91.0^f^	1200 or 2400 or 4800	Continuous	Placebo	/	Continuous	24	24
PREVENT2 [31]^g^	2014	592	56.1 (11.0)	√	√	Acute uncomplicated diverticulitis	Remission	59.7	115.5^f^	1200 or 2400 or 4800	Continuous	Placebo	/	Continuous	24	24
SAG-37 [32]^h^	2017	345	58.6 (9.3)		√	Acute uncomplicated diverticulitis	Remission	55.0	87.0 (48.0)	3000	Continuous	Placebo	/	Continuous	12	12
SAG-51 [32]^h^	2017	330	55.4 (10.6)		√	Acute uncomplicated diverticulitis	Remission	51.2	89.0 (44.0)	1500 or 3000	Continuous	Placebo	/	Continuous	24	24

SD, standard deviation; na, not assessed.

^a^The study includes 3 arms (mesalazine, probiotic, and mesalazine plus probiotic), but only the mesalazine and probiotic arms were considered in this analysis.

^b^8 participants were included in both studies.

^c^96 participants were randomized; 4 were excluded for no study drug assumption, but there is no mention on their study group. Thus, only 92 participants are included in our analysis.

^d^The study includes 3 arms (mesalazine, placebo, and mesalazine plus probiotic), but only the mesalazine and placebo arms were considered in this analysis.

^e^The study includes 4 arms (mesalazine, probiotic, mesalazine plus probiotic, and placebo), but only the mesalazine, probiotic, and placebo arms were considered in this analysis.

^f^Median value.

^g^The same publication includes two randomized trials with identical study design.

^h^The same publication includes two randomized trials.

**Table 2:** Summary of findings: mesalazine versus control interventions by subtype of diverticular disease.

**Outcome**	**Subtype of diverticular disease**	**No. of participants (no. of studies)**	**Absolute effect ** **(per 100 patients treated)**	**Relative effect ** **(95**%** CI)**	**Certainty of the evidence (GRADE)**	**Conclusion**
Achievement of disease remission	Symptomatic uncomplicated diverticular disease	123 (1)	3 more per 100 (95% CI: 14 fewer to 19 more)	1.04 (0.81 to 1.34)	Very Low ●○○○	It is uncertain whether mesalazine may lead to no difference in the achievement of disease remission.
	Acute uncomplicated diverticulitis	81 (1)	20 more per 100 (95% CI: 3 fewer to 38 more)	2.67 (1.05 to 6.79)	Very Low ●○○○	It is uncertain whether mesalazine may lead to the achievement of disease remission.
Disease recurrence	Symptomatic uncomplicated diverticular disease	216 (2)	13 fewer per 100 (95% CI: 23 to 2 fewer)	0.52 (0.28 to 0.97)	Low ●●○○	Mesalazine may decrease disease recurrence.
	Acute uncomplicated diverticulitis	2196 (7)	3 fewer per 100 (95% CI: 12 fewer to 6 more)	0.90 (0.61 to 1.33)	Low ●●○○	Mesalazine may lead to no difference in disease recurrence.
Acute diverticulitis onset in symptomatic uncomplicated diverticular disease	Symptomatic uncomplicated diverticular disease	484 (3)	3 fewer per 100 (95% CI: 7 fewer to 0)	0.26 (0.06 to 1.20)	Low ●●○○	Mesalazine may lead to no difference in the development of acute diverticulitis in patients with symptomatic uncomplicated diverticular disease.
Need for surgery	Symptomatic uncomplicated diverticular disease	424 (2)	No effect per 100 (95% CI: 1 fewer to 1 more)	0.68 (0.03 to 16.39)	Very Low ●○○○	It is uncertain whether mesalazine may lead to no difference in the need for surgery.
	Acute uncomplicated diverticulitis	1263 (3)	1 more per 100 (95% CI: 0 to 2 more)	1.41 (0.51 to 3.90)	Low ●●○○	Mesalazine may lead to no difference in the need for surgery.
Any adverse events	Symptomatic uncomplicated diverticular disease	391 (2)	1 fewer per 100 (95% CI: 5 fewer to 3 more)	1.04 (0.55 to 1.98)	Low ●●○○	Mesalazine may lead to no difference in any adverse events.
	Acute uncomplicated diverticulitis	2196 (7)	3 more per 100 (95% CI: 2 fewer to 7 more)	1.03 (0.96 to 1.11)	Moderate ●●●○	Mesalazine probably results in no difference in any adverse events.
All-cause mortality	Symptomatic uncomplicated diverticular disease	607 (4)	No effect per 100 (95% CI: 1 fewer to 1 more)	No event in included studies	Very Low ●○○○	It is uncertain whether mesalazine may lead to no difference in all-cause mortality.
	Acute uncomplicated diverticulitis	1512 (5)	No effect per 100 (95% CI: 1 fewer to 1 more)	0.52 (0.05 to 5.68)	Very Low ●○○○	It is uncertain whether mesalazine may lead to no difference in all-cause mortality.
Diverticular disease related mortality	Symptomatic uncomplicated diverticular disease	607 (4)	No effect per 100 (95% CI: 1 fewer to 1 more)	No event in included studies	Very Low ●○○○	It is uncertain whether mesalazine may lead to no difference in diverticular disease related mortality.
	Acute uncomplicated diverticulitis	1512 (5)	No effect per 100 (95% CI: 1 fewer to 1 more)	No event in included studies	Very Low ●○○○	It is uncertain whether mesalazine may lead to no difference in diverticular disease related mortality.
Global symptoms score^a^	Symptomatic uncomplicated diverticular disease	326 (2)	Standardized mean difference: 1.01 lower (95% CI: 1.51 to 0.52 lower)	-	Low ●●○○	Mesalazine may improve the global symptoms score.
	Acute uncomplicated diverticulitis	153 (2)	Standardized mean difference: 0.56 lower (95% CI: 0.88 to 0.24 lower)	-	Low ●●○○	Mesalazine may slightly improve the global symptoms score.
Time to recurrence (days)	Symptomatic uncomplicated diverticular disease	- (-)	No studies	-	Absent	No studies were found that evaluated the impact of mesalazine on time to disease recurrence of disease.
	Acute uncomplicated diverticulitis	91 (3)	Average difference in days: 30 lower (95% CI: 55 to 5 lower)	-	Very Low ●○○○	It is uncertain whether mesalazine may lead to a decrease in time to disease recurrence.

^a^The standardized mean difference was used because global symptoms were assessed using different scales across included studies: 0 to 33 [24], 0 to 36 [25], 0 to 60 [29], and 4 to 40 [28].

**Table 3:** Quality of life assessment of people enrolled in randomized trials comparing mesalazine with control interventions for diverticular disease.

**Study, year (reference)**	**Quality-of-life instrument**	**Validation of the instrument**	**Time of assessment**	**Method of Reporting**	**Domains reported**	**Results**
Comparato et al., 2007*a* [24]	Medical Outcome Study (MOS) 36-Item Short-Form Health Survey (SF-36, Italian version 1.6) questionnaire	Yes^a^	Baseline and 6 months	Mean change of each domain score between baseline and 6 months in the mesalazine and rifaximin group. Difference of the mean scores of each domain at baseline and 6 months between the two groups.	Physical health:	In the mesalazine group there was a significant improvement of physical functioning (p=0.05), role limitation-physical (p=0.034), general health (p=0.01), and social functioning (p<0.01) at 6 months. In the rifaximin group there was a significant improvement of role limitation-physical (p=0.04) and social functioning (p=0.03) at 6 months. There was no statistically significant difference of SF-36 mean scores between the two groups at baseline. There was a higher improvement of physical functioning (p<0.05) and general health (p=0.01) in the mesalazine than rifaximin group at 6 months.
					Physical functioning	
					Role limitation-physical	
					Bodily	
					pain	
					General health	
					Mental health:	
					Role limitation-emotional	
					Vitality	
					Mental health	
					Social functioning	
Parente et al., 2013 [28]	Therapy Impact Questionnaire (TIQ), quality-of-life sub-score^b^	No	Baseline and every 3 months until 24 months	Difference of the mean quality-of-life sub-scores at baseline and 24 months between mesalazine and placebo group.	Sleeping problem Physical status interfering with sexual activity Problem in out-door activities Problem in social activities Problem in in-house activities different from house works Problem in daily work activities Problem in free-time activities	There was no statistically significant difference of the total quality-of-life sub-score between the mesalazine and placebo group at baseline and 24 months.
PREVENT1, 2014 [31]	(1) EuroQol five dimensions questionnaire (EQ-5D) (2) Health Utilities Index Version Mark 2 (HUI2) questionnaire	Yes^a^	Baseline and 16, 52, 78, 104 weeks	Difference of the total EQ-5D and HUI2 scores at baseline and 104 weeks across study arms (mesalazine 1200mg, mesalazine 2400mg, mesalazine 4800mg, placebo).	EQ-5D questionnaire:	There was no statistically significant difference of the total EQ-5D and HUI2 scores at baseline and 104 weeks across study arms.
					Mobility	
					Self-care	
					Usual activities	
					Pain/discomfort	
					Anxiety/depression	
					HUI2 questionnaire:	
					Sensation	
					Cognition	
					Mobility	
					Self-care	
					Emotion	
					Pain	
					Fertility	
PREVENT2, 2014 [31]	(1) EuroQol five dimensions questionnaire (EQ-5D) (2) Health Utilities Index Version Mark 2 (HUI2) questionnaire	Yes^a^	Baseline and 16, 52, 78, 104 weeks	Difference of the total EQ-5D and HUI2 scores at baseline and 104 weeks across study arms (mesalazine 1200mg, mesalazine 2400mg, mesalazine 4800mg, placebo).	EQ-5D questionnaire:	There was no statistically significant difference of the total EQ-5D and HUI2 scores at baseline and 104 weeks across study arms.
					Mobility	
					Self-care	
					Usual activities	
					Pain/discomfort	
					Anxiety/depression	
					HUI2 questionnaire:	
					Sensation	
					Cognition	
					Mobility	
					Self-care	
					Emotion	
					Pain	
					Fertility	

EQ-5D, EuroQol five dimensions; HUI2, Health Utilities Index Version Mark 2.

^a^Instrument validated to measure generic health outcomes in all therapeutic areas.

^b^11-item questionnaire: the sum of items 1-4 defines the physical condition subscore, while the sum of items 5-11 defines the quality-of-life subscore.

## Supplementary Materials

Supplementary Materials Supplementary Table 1: recommendations from international guideline agencies about therapeutic options in diverticular disease. Supplementary Table 2: search strategies in MEDLINE, EMBASE, CENTRAL, and ClinicalTrials.gov. Supplementary Table 3: comparative effectiveness of mesalazine versus control interventions by subtype of diverticular disease. Supplementary Table 4: symptoms assessment of participants enrolled in randomized trials comparing mesalazine with control interventions for diverticular disease. Supplementary Table 5: comparative effectiveness of mesalazine versus control interventions by type of control intervention, stratified by subtype of diverticular disease. Supplementary Table 6: comparative effectiveness of mesalazine versus control interventions by follow-up duration, stratified by subtype of diverticular disease. Supplementary Table 7: comparative effectiveness of mesalazine versus control interventions by dose of mesalazine, stratified by subtype of diverticular disease. Supplementary Table 8: comparative effectiveness of mesalazine versus control interventions by mode of administration of mesalazine, stratified by subtype of diverticular disease. Supplementary Figure 1: (a) risk of bias graph: review authors' judgement about each risk of bias item presented as percentages across all included studies; (b) risk of bias summary: review authors' judgement about each risk of bias item for each included study.
