# Supplementary material for: Mesalazine for People with Diverticular Disease: A Systematic Review of Randomized Controlled Trials
Source: Can J Gastroenterol Hepatol. 2018 Sep 16;2018:5437135. doi: 10.1155/2018/5437135 (PMC6167594; doi:10.1155/2018/5437135)
Supplement: Supplementary Materials — Supplementary Table 1: recommendations from international guideline agencies about therapeutic options in diverticular disease. Supplementary Table 2: search strategies in MEDLINE, EMBASE, CENTRAL, and ClinicalTrials.gov. Supplementary Table 3: comparative effectiveness of mesalazine versus control interventions by subtype of diverticular disease. Supplementary Table 4: symptoms assessment of participants enrolled in randomized trials comparing mesalazine with control interventions for diverticular disease. Supplementary Table 5: comparative effectiveness of mesalazine versus control interventions by type of control intervention, stratified by subtype of diverticular disease. Supplementary Table 6: comparative effectiveness of mesalazine versus control interventions by follow-up duration, stratified by subtype of diverticular disease. Supplementary Table 7: comparative effectiveness of mesalazine versus control interventions by dose of mesalazine, stratified by subtype of diverticular disease. Supplementary Table 8: comparative effectiveness of mesalazine versus control interventions by mode of administration of mesalazine, stratified by subtype of diverticular disease. Supplementary Figure 1: (a) risk of bias graph: review authors' judgement about each risk of bias item presented as percentages across all included studies; (b) risk of bias summary: review authors' judgement about each risk of bias item for each included study. [file 5437135.f1.pdf]

Supplementary Table 1. Recommendations from international guideline agencies about therapeutic options in diverticular disease.

Agency (reference)	Type of Document: Guide-line	Type of Document: Consensus paper	Type of Document: Position state-ment	Year	Subtype of diverticular disease addressed in the recommendation	Recommendation	Quality of evidence	Strength of recommen-dation	Tool used for assessing evidence
American College of Gastro-enterology (9)	√			1999	Acute uncomplicated diverticulitis	• Selected patients with mild diverticulitis can be treated as outpatients with broad-spectrum oral antibiotics.<br>• More severe illness or comorbid disease should be treated with bowel rest and intravenous antibiotics.<br>• Elective surgery may be reasonable in patients with recurrent attacks.	Not Assessed	Not Assessed	None
					Acute complicated diverticulitis	• Small pericolic abscesses can be managed with antibiotics and bowel rest; larger abscesses require drainage.<br>• Multiloculated, inaccessible or poorly responsive abscesses may require initial surgical drainage.<br>• Diverticular fistulas are generally managed surgically.<br>• Acute obstruction is usually self-limited and responds well to conservative therapy. Symptomatic chronic strictures may be managed endoscopically or surgically.<br>• Angiography and colonoscopy may be useful in ongoing bleeding, if unsuccessful surgery may be required.	Not Assessed	Not Assessed	
European Association for Endoscopic Surgery (3)		√		1999	Acute uncomplicated diverticulitis	• Conservative treatment is indicated in cases with a first attack.<br>• In mild cases it consists of oral hydration, oral antibiotics and antispasmodics.<br>• In moderate or severe cases, oral feeding should be stopped to allow bowel rest. Hydration and antibiotics should be given intravenously. Analgesics can be given as required.<br>• Patients who are not suffering from an acute attack should maintain a diet high in fiber.<br>• Patients should be considered for elective surgery if they have had at least two attacks of the disease.	Not Assessed	Not Assessed	US Agency for Health Care Policy and Research
					Acute complicated diverticulitis	• Complications such as colovesicular or colovaginal fistulas, stenoses, and bleeding are indications for operation.	Not Assessed	Not Assessed	
World Gastroenterology Organisation (10)	√			2007	Acute uncomplicated diverticulitis	• Outpatient with mild abdominal pain/tenderness and no systemic symptoms: acute low-residue diet and antibiotics.<br>• Inpatient with severe signs/symptoms: ensure bowel rest, intravenous antibiotics and fluids, analgesia.<br>• Indication for elective surgery: two or more episodes of diverticulitis severe enough to cause hospitalization.	Not Assessed	Not Assessed	None
					Acute complicated diverticulitis	• Urgent surgical intervention is mandatory if complications occur.	Not Assessed	Not Assessed	
Association of Coloproctology of Great Britain and Ireland (11) [a]			√	2011	Acute uncomplicated diverticulitis	• The majority of patients can be managed with a medical approach in the longer term.<br>• The decision on elective resection should be made on an individual basis.	III	C	US Agency for Health Care Policy and Research [b]
					Acute complicated diverticulitis	• The laparoscopic approach is appropriate. It may confer benefits to patient recovery.	III	D	
Danish Surgical Society (8)	√			2012	Symptomatic uncomplicated diverticular disease	• Cyclic rifaximin plus fibre may have a place in the therapeutic armamentarium.<br>• In chronic disease or by frequent relapse, resection can be considered if the condition is intolerable.	Ia<br>III	A<br>C	Danish Colorectal Cancer Group classification system [c]
					Acute uncomplicated diverticulitis	• Antibiotics are not routinely recommended.<br>• Antibiotics should still be used for septicaemia, affected general condition, pregnancy or immunosuppression.<br>• Dietary restriction and bed rest is unproven.<br>• Any recommendation for routine resection following multiple cases of diverticulitis must await RCTs results.	Ib<br>IV<br>/<br>/	A<br>C<br>/<br>C	
					Acute complicated diverticulitis	• Abscesses suitable for drainage are recommended drained under US- or CT-guidance combined with antibiotics.<br>• Abscesses not suitable for drainage are treated conservatively with antibiotics under clinical observation.<br>• Abscesses treatment failures are handled surgically.<br>• In diverticulitis with fistula or stenosis resection is recommended if the patient's condition allows this.	III	C	

Agency (reference)	Type of Document: Guide-line	Type of Document: Consensus paper	Type of Document: Position state-ment	Year	Subtype of diverticular disease addressed in the recommendation	Recommendation	Quality of evidence	Strength of recommen-dation	Tool used for assessing evidence
Netherlands Society of Surgery (12)	√			2013	Acute uncomplicated diverticulitis	• There is no evidence that antibiotics should be routinely administered.<br>• Antibiotic treatment is recommended when signs of generalized infection and affected general condition or signs of bacteremia or septicemia are present. Antibiotic treatment is recommended in immunocompromised patients.<br>• Following an attack of diverticulitis give lifestyle advice (daily fiber intake, weight reduction, cessation of smoking and increasing physical activity).<br>• The combination of mesalzine and rifaximin is more effective than rifaximin alone in preventing recurrences.<br>• The combination of probiotics and anti-inflammatory drugs is preferred over probiotics alone in preventing recurrences<br>• Patient-related factors, not the number of previous episodes, should play the most important role in selecting patients who might benefit from elective sigmoid resection.	2<br>4<br>4<br>2<br>2<br>3	/<br>/<br>/<br>/<br>/<br>/	Dutch classification system for evidence-based guideline develop-ment [d]
					Acute complicated diverticulitis	• Smaller abscesses (<4–5 cm) can be treated with antibiotics alone, whereas larger abscesses can best be treated with percutaneous drainage combined with antibiotic treatment.<br>• Operative treatment is considered standard therapy for patients with Hinchey III and IV.	3<br>/	/<br>/	
Task Force of the American Society of Colon and Rectal Surgeons (13)	√			2014	Acute uncomplicated diverticulitis	• Nonoperative treatment typically includes oral or intravenous antibiotics and diet modification.<br>• The decision to recommend elective sigmoid colectomy after recovery should be individualized.<br>• Urgent sigmoid colectomy is required if nonoperative management of acute diverticulitis fails.	Low<br>Moderate<br>Moderate	Strong<br>Strong<br>Strong	GRADE
					Acute complicated diverticulitis	• Patients require hospital admission and, typically, intravenous antibiotics and bowel rest.<br>• Image-guided percutaneous drainage is the most appropriate treatment for stable patients with large abscesses.<br>• Urgent sigmoid colectomy is required for patients with diffuse peritonitis.<br>• Elective colectomy should typically be considered after the patient recovers from an acute episode.	/<br>Moderate<br>Moderate<br>Moderate	/<br>Strong<br>Strong<br>Strong	
Italian Group on Diverticular Diseases (4)		√		2014	Symptomatic uncomplicated diverticular disease	• Fibre plus rifaximin provide a greater prevalence of symptom-free patients compared to fibre alone.<br>• Rifaximin plus fibre is more effective than fibre alone in preventing acute diverticulitis (low therapeutic advantage).<br>• There is no clear evidence that mesalazine alone is effective in reducing symptoms.<br>• There is no clear evidence that mesalazine reduces acute episodes of diverticulitis.<br>• There is no clear evidence that mesalazine reduces recurrences.<br>• There is insufficient evidence that probiotics are effective in reducing symptoms.	2b<br>2b<br>2b<br>3b<br>2b<br>4	B<br>B<br>B<br>C<br>B<br>C	Oxford Centre for Evidence-Based Medicine [e]
					Acute uncomplicated diverticulitis	• Antibiotics may not improve outcome and are used on a case-by-case basis.<br>• There is no clear evidence that mesalazine alone is effective in reducing symptoms.<br>• There is no clear evidence that mesalazine reduces recurrences.<br>• The decision to perform elective resection after one or more episodes should be undertaken case-by-case.	3b<br>2b<br>3b<br>2b	C<br>B<br>C<br>B	
					Acute complicated diverticulitis	• Hospitalization, bowel rest and broad-spectrum antibiotics are needed.<br>• Elective surgery should be recommended.<br>• The best treatment for a diverticular abscess >4 cm is percutaneous guided drainage. Diverticular abscesses not responding, or not amenable, to non-operative management should be treated surgically.	3b<br>3a<br>3b	C<br>B<br>C	

Agency (reference)	Type of Document			Year	Subtype of diverticular disease addressed in the recommendation	Recommendation	Quality of evidence	Strength of recommen-dation	Tool used for assessing evidence
	Guide-line	Consensus paper	Position state-ment						
German Society for Gastroenterology Digestive and Metabolic Diseases and German Society for General and Visceral Surgery (14)	√			2014	Symptomatic uncomplicated diverticular disease	• Painful uncomplicated diverticular disease can be treated with mesalazine (orally).	/	OR	Tool created by authors for this guideline [f]
					Acute uncomplicated diverticulitis	• Antibiotic therapy can be omitted subject to close clinical monitoring.<br>• Antibiotic therapy should be given to patients with risk indicators of complicated course.<br>• If conservative therapy does not result in a cure, surgical therapy should be considered.<br>• A recommendation for prophylaxis of recurrences with drugs (mesalazine, rifaximin, probiotics) cannot be given.<br>• It should be operated after a careful risk/benefit assessment depending on the clinical presentation and not on the number of previous episodes.	/<br>/<br>/<br>/<br>/	OR<br>R<br>R<br>OR<br>R	
					Acute complicated diverticulitis	• Oral nutrition can be given depending on the clinical situation.<br>• Antibiotic therapy must be administered.<br>• Retroperitoneal or paracolic abscesses can be drained interventionally (US, CT).<br>• Abscesses not amenable to drainage or not responding within 72h to conservative treatment should undergo surgery.<br>• Patients with free perforation and peritonitis must undergo surgery immediately after diagnosis.<br>• Fistulas and clinically relevant stenosis should undergo surgery.<br>• Where there is a source of bleeding, endoscopic hemostasis must be attempted. If not possible, angiography can be performed. In all other cases of persistent bleeding surgical therapy must be undertaken urgently.	/<br>/<br>/<br>/<br>/<br>/<br>/	OR<br>SR<br>R<br>R<br>SR<br>R<br>SR	
Italian Society of Colon and Rectal Surgery (7)	√			2015	Symptomatic uncomplicated diverticular disease	• In the treatment we suggest that some patients may benefit of rifaximin with fibre and some from mesalazine alone.<br>• Rifaximin plus fibre is more effective than fibre alone in preventing acute diverticulitis (low therapeutic advantage).<br>• There is insufficient evidence that treatment with probiotics is effective in reducing symptoms.	Moderate<br>Moderate<br>Moderate	Strong<br>Strong<br>Conditional	GRADE
					Acute uncomplicated diverticulitis	• Antibiotic use on a case-by-case basis should possibly be considered.<br>• There is no substantial evidence that mesalazine alone is effective in preventing recurrence.<br>• We recommend that the decision of elective resection after one or more episodes should be undertaken “case by case”.<br>• We suggest urgent surgery for diffuse peritonitis and failure to improve despite appropriate medical therapy.	Moderate<br>Moderate<br>Low<br>Moderate	Strong<br>Strong<br>Strong<br>Conditional	
					Acute complicated diverticulitis	• Elective surgery should be recommended to patients with complicated disease (fistula, stenosis).<br>• The best treatment for a diverticular abscess >4 cm is percutaneous guided drainage. Diverticular abscesses not responding, or not amenable, to non-operative management should be treated surgically.	Low<br>High	Strong<br>Conditional	
American Gastroenterolo-gical Association (15)	√			2015	Acute uncomplicated diverticulitis	• Antibiotics should be used selectively, rather than routinely.<br>• The AGA suggests against elective colonic resection after an initial episode. The decision should be individualized.<br>• The AGA suggests a fiber-rich diet or fiber supplementation in patients with a history of acute diverticulitis.<br>• The AGA recommends against the use of mesalazine after an episode of acute diverticulitis.<br>• The AGA suggests against the use of rifaximin after an episode of acute diverticulitis.<br>• The AGA suggests against the use of probiotics after an episode of acute diverticulitis.	Low<br>Very-low<br>Very-low<br>Moderate<br>Very-low<br>Very-low	Conditional<br>Conditional<br>Conditional<br>Strong<br>Conditional<br>Conditional	GRADE
World Society of Emergency Surgery (16)	√			2016	Acute uncomplicated diverticulitis	• Antimicrobial therapy can be avoided in immunocompetent patients without systemic manifestations of infection.<br>• If patients need antimicrobial therapy, oral administration may be acceptable.<br>• Patient-related factors and not number of previous episodes of diverticulitis, should be considered in planning elective sigmoid resection in patients treated conservatively	High<br>Moderate<br>Low	Strong<br>Strong<br>Strong	GRADE
					Acute complicated diverticulitis	• Patients with small diverticular abscesses (<4-5 cm) may be treated by antibiotics alone.<br>• Patients with large abscesses (>4–5 cm) can best be treated by percutaneous drainage with antibiotic treatment.<br>• Patients with CT findings of distant air without diffuse fluid may be treated by conservative treatment in selected cases<br>• If the conservative treatment fails in patients with distant air without diffuse fluid, surgical resection is suggested.<br>• Surgical resection is advised for managing diffuse peritonitis.	Low<br>Low<br>Low<br>Moderate<br>Moderate	Strong<br>Strong<br>Strong<br>Strong<br>Strong	

[a] This position statement focuses on surgical therapy for diverticular disease.

[b] Level III= evidence obtained from well-designed nonexperimental descriptive studies, such as comparative studies, correlation studies and case studies; Grade C= evidence of type IIa (evidence obtained from at least one well-designed controlled study without randomization), IIb (evidence obtained from at least one other well-designed quasi-experimental study) or III but inconsistent findings.

[c] Level Ia= meta-analysis of RCTs; Level Ib= at least one RCT; Level III= good descriptive studies (cohort, case control and case series); Level IV= expert committees, esteemed Authorities, cases; Grade A= at least one RCT among several good studies, all of which are fundamental to the recommendation (Ia, Ib); Grade C= requires expert committee or authority, but says there are no good clinical studies as a basis.

[d] Level 2= one study with evidence level A2 (double blind RCT of good study quality with an adequate number of study participants) or at least 2 independent studies with evidence level B (comparative studies, but without all the features mentioned for level A2, including patient-control studies, cohort studies); Level 3= one study with evidence level B or level C (noncomparative studies); Level 4= expert opinion.

[e] Level 2b= individual cohort study (including low quality RCT; e.g., <80% follow-up); Level 3a= systematic review (with homogeneity) of case-control studies; Level 3b= individual case-control study; Level 4= case-series and poor quality cohort and case-control studies; Grade B= consistent level 2 or 3 studies or extrapolations from level 1 (systematic review of RCT or individual RCT with narrow confidence interval) studies; Grade C= level 4 studies or extrapolations from level 2 or 3 studies.

[f] Strength of recommendation graded as: strong recommendation (SR), recommendation (R), open recommendation (OR), negative recommendation (NR), strongly negative recommendation (SNR).

Supplementary Table 2. Search Strategies in MEDLINE, EMBASE, the Cochrane Central Register of Controlled Trials and ClinicalTrials.gov.

Ovid MEDLINE® Epub Ahead of Print, In-Process & Other Non-Indexed Citations, Ovid MEDLINE® Daily and Ovid MEDLINE® 1946 to July 31st 2018

#	Searches	Results
1	Diverticulitis/	2786
2	exp Diverticulosis, Colonic/	3803
3	Diverticulum/	8798
4	(diverticulitis or diverticula* or diverticulosis or diverticulum).tw.	25057
5	or/1-4	27721
6	Mesalamine/	3179
7	Sulfasalazine/	3998
8	mesalamine.tw.	857
9	mesalazine.tw.	1303
10	5 aminosalicylic acid.tw.	1681
11	5 ASA.tw.	1405
12	5ASA.tw.	54
13	sulfasalazine.tw.	2938
14	sulphasalazine.tw.	1171
15	salazosulfapyridine.tw.	246
16	balsalazide.tw.	102
17	or/6-16	9818
18	and/5,17	163
19	randomized controlled trial.pt.	467579
20	controlled clinical trial.pt.	94272
21	pragmatic clinical trial.pt.	602
22	randomized.ab.	409577
23	placebo.ab.	190813
24	drug therapy.fs.	2011552
25	randomly.ab.	284046
26	trial.ab.	429215
27	groups.ab.	1748297
28	Cross-over Studies/	42589
29	(crossover or cross-over).tw.	75938
30	or/19-29	4168581
31	animals/ not (humans/ and animals/)	4391739
32	30 not 31	3605465
33	and/18,32	103
34	Remove duplicates from 33	100

EMBASE 1996 to 2018 Week 31 (searched July 31st 2018)

#	Searches	Results
1	Diverticulitis/	5151
2	Diverticulosis/	7274
3	Colon Diverticulosis/	3639
4	Intestine Diverticulosis/	716
5	(diverticulitis or diverticula* or diverticulosis or diverticulum).tw.	18718
6	or/1-5	22949
7	Mesalazine/	13666
8	Salazosulfapyridine/	16913
9	Balsalazide/	795
10	mesalazine.tw.	1961
11	mesalamine.tw.	1663
12	5 aminosalicylic acid.tw.	1544
13	5 ASA.tw.	2239
14	5ASA.tw.	199
15	sulfasalazine.tw.	3518
16	sulphasalazine.tw.	771
17	salazosulfapyridine.tw.	205
18	balsalazide.tw.	168
19	or/7-18	29135
20	and/6,19	441
21	randomized controlled trial/	411986
22	crossover procedure/	47883
23	double-blind procedure/	113806
24	single-blind procedure/	26706
25	random$.tw.	1075391
26	factorial$.tw.	26580
27	(crossover$ or cross-over$).tw.	68171
28	placebo$.tw.	207431
29	(double$ adj blind$).tw.	132564
30	(singl$ adj blind$).tw.	16182
31	assign$.tw.	272154
32	allocat$.tw.	103467
33	or/21-32	1464300
34	and/20,33	96

CENTRAL from inception to Issue 6 of 12, June 2018 (searched July 31st 2018)

ID	Search	Results
#1	diverticulitis:ti,ab,kw	343
#2	diverticulosis:ti,ab,kw	191
#3	diverticula*:ti,ab,kw	193
#4	diverticulum:ti,ab,kw	115
#5	{or #1-#4}	608
#6	mesalamine:ti,ab,kw	566
#7	mesalazine:ti,ab,kw	652
#8	sulfasalazine:ti,ab,kw	679
#9	sulphasalazine:ti,ab,kw	252
#10	salazosulfapyridine:ti,ab,kw	551
#11	"5 aminosalicylic acid":ti,ab,kw	259
#12	"5 ASA":ti,ab,kw	269
#13	"5ASA":ti,ab,kw	15
#14	balsalazide:ti,ab,kw	41
#15	{or #6-#14}	1943
#16	{and #5, #15}	45
#17	Remove duplicates form #16	40

ClinicalTrials.gov (searched July 31st 2018)

ID	Search (keywords)	Results
#1	mesalamine AND (diverticulitis OR diverticular)	9

Supplementary Table 3. Comparative effectiveness of mesalazine versus control interventions by subtype of diverticular disease.

**Outcome analyzed, subtype of diverticular disease (reference)**	**Studies reporting outcome, *n***	**Patients enrolled, *n***	**Risk Ratio (95% Confidence Interval)** [a]	**Heterogeneity**		**Test for subgroup differences, p value**
				**Chi-square**	**I^2, %**	
Number of patients achieving disease remission [b]						0.06
Symptomatic uncomplicated diverticular disease(27)	1	123	1.04 (0.81 to 1.34)	-	-	
Acute uncomplicated diverticulitis(29)	1	81	2.67 (1.05 to 6.79)	-	-	
Total	2	204	1.51 (0.57 to 3.98)	4.18	76	
Number of patients with recurrence of disease						0.31
Symptomatic uncomplicated diverticular disease(23, 30)	2	216	0.52 (0.28 to 0.97)	0.26	0	
Acute uncomplicated diverticulitis(22, 28, 29, 31, 32)	7	2196	0.90 (0.61 to 1.33)	25.02	76	
Total	9	2412	0.83 (0.58 to 1.19)	25.39	76	
Number of patients developing acute diverticulitis in symptomatic uncomplicated diverticular disease(23, 25, 30)	3	484	0.26 (0.06 to 1.20)	0.30	0	
Number of patients needing surgery						0.67
Symptomatic uncomplicated diverticular disease(25, 30) [c]	2	424	0.68 (0.03 to 16.39)	-	-	
Acute uncomplicated diverticulitis(29, 31)	3	1263	1.41 (0.51 to 3.90)	0.97	0	
Total	5	1687	1.32 (0.50 to 3.48)	1.15	0	
Number of patients needing hospitalization						-
Symptomatic uncomplicated diverticular disease(23)	1	60	0.33 (0.01 to 7.87)	-	-	
Acute uncomplicated diverticulitis	-	-	-	-	-	
Total	1	60	0.33 (0.01 to 7.87)	-	-	
Number of patients with any adverse events						0.97
Symptomatic uncomplicated diverticular disease(25, 27)	2	391	1.04 (0.55 to 1.98)	0.71	0	
Acute uncomplicated diverticulitis(22, 28, 29, 31, 32)	7	2196	1.03 (0.96 to 1.11)	8.34	28	
Total	9	2587	1.03 (0.97 to 1.10)	9.05	12	
All-cause mortality						-
Symptomatic uncomplicated diverticular disease(23, 25, 27, 30) [d]	4	607	-	-	-	
Acute uncomplicated diverticulitis(22, 29, 31, 32) [e]	5	1512	0.52 (0.05 to 5.68)	-	-	
Total	9	2119	0.52 (0.05 to 5.68)	-	-	
Diverticular disease related mortality						-
Symptomatic uncomplicated diverticular disease(23, 25, 27, 30) [d]	4	607	-	-	-	
Acute uncomplicated diverticulitis(22, 29, 31, 32) [d]	5	1512	-	-	-	
Total	9	2119	-	-	-	

**Outcome analyzed, subtype of diverticular disease (reference)**	**Studies reporting outcome, *n***	**Patients enrolled, *n***	**Mean Difference (95% Confidence Interval)**	**Heterogeneity**		**Test for subgroup differences, p value**
				**Chi-square**	**I^2, %**	
Time to recurrence (days) [f]						-
Symptomatic uncomplicated diverticular disease	-	-	-	-	-	
Acute uncomplicated diverticulitis(28, 29, 32)	3	91	-30.04 (-55.18 to -4.90)	2.36	15	
Total	3	91	-30.04 (-55.18 to -4.90)	2.36	15	
Time to remission						-
Symptomatic uncomplicated diverticular disease	-	-	-	-	-	
Acute uncomplicated diverticulitis	-	-	-	-	-	
Time to acute diverticulitis development in symptomatic uncomplicated diverticular disease						-
Symptomatic uncomplicated diverticular disease	-	-	-	-	-	
Acute uncomplicated diverticulitis	-	-	-	-	-	
Time to surgery						-
Symptomatic uncomplicated diverticular disease	-	-	-	-	-	
Acute uncomplicated diverticulitis	-	-	-	-	-	

[a] values <1 or >1 indicate the direction of effect for mesalazine or control interventions, respectively.
[b] values >1 or <1 indicate the direction of effect for mesalazine or control interventions, respectively.
[c] no event was reported in one included study (25).
[d] no event was reported in included studies.
[e] no event was reported in four included studies: Stollman et al. (29), PREVENT1(31), SAG-37(32) and SAG-51(32).
[f] values >0 or <0 indicate the direction of effect for mesalazine or control interventions, respectively.

Supplementary Table 4. Symptoms assessment of participants enrolled in randomized trials comparing mesalazine with control interventions for diverticular disease.

Study, year (reference)	Symptom assessment instrument	Validation of the instrument	Time of assessment	Method of Reporting	Symptoms reported	Results
Trespi et al., 1999 (22)	Frequency and duration of symptoms	No	From baseline to 48 months	Difference in symptom frequency or duration at 48 months between the mesalazine and no treatment group.	Weekly frequency of bowel movements, abdominal pain, abdominal gaseous distension and/or fever, any other symptoms.	The mean duration of abdominal pain at diverticular disease exacerbation was significantly shorter in mesalazine than no treatment group (p=0.002), while there were no significant differences between the two groups with regard to the remaining symptom scores.
Tursi et al., 2006 (23)	Visual 0 to 10 points scales	No	Baseline and 1, 2, 6, 9 and12 months	Difference in total symptomatic score at 12 months between the mesalazine and Lactobacillus casei subspecies DG group.	Constipation, diarrhea, abdominal pain, rectal bleeding, mucus with the stools.	All participants were asymptomatic at baseline (score=0 for all symptoms).<br>There was a statistically significant lower total symptomatic score in mesalazine than Lactobacillus casei subspecies DG group at 12 months (p<0.001).
Comparato et al., 2007*a* (24)	11-items global symptomatic score (GSS), using visual 0 to 3 points scales	No	Baseline and 6 months	Mean change of GSS between baseline and 6 months in the mesalazine and rifaximin group.<br>Difference of mean GSS at baseline and 6 months between the two groups.	Upper abdominal pain/discomfort, lower abdominal pain/discomfort, bloating, tenesmus, diarrhea, abdominal tenderness, fever, general illness, nausea, emesis, dysuria.	In the mesalazine group there was a statistically significant reduction of mean GSS (p<0.001) at 6 months.<br>In the rifaximin group there was a statistically significant reduction of mean GSS (p<0.01) at 6 months.<br>There was no statistically significant difference of mean GSS between the two groups at baseline. There was a significant higher reduction of mean GSS in the mesalazine than rifaximin group at 6 months (p=0.019).
Comparato et al., 2007*b* (25)	12-items global symptomatic score (GSS), using visual 0 to 3 points scales	No	Baseline and every 3 months until 12 months	Mean change of GSS between baseline and 6 months as well as baseline and 12 months in the mesalazine and rifaximin group.<br>Difference of mean GSS at baseline, 6 and 12 months between the two groups.	Upper abdominal pain/discomfort, lower abdominal pain/discomfort, bloating, tenesmus, diarrhea, abdominal tenderness, fever, general illness, nausea, emesis, dysuria, bleeding.	In both groups, there was a statistically significant reduction of mean GSS at 6 and 12 months (p<0.001).<br>There was no statistically significant difference of mean GSS between the two groups at baseline. There was a significant higher reduction of mean GSS in the mesalazine than rifaximin group at 6 and 12 months (p<0.001).
Smith et al., 2012 (26)	Hours per day	No	Baseline and 12 weeks	Median change of symptom duration between baseline and 12 weeks in the mesalazine and placebo group	Abdominal pain and bowel habit.	There was a statistically significant reduction of the duration of abdominal pain only in the mesalazine group (p=0.041).
Kruis et al., 2013 (27)	100 mm visual analogue scoring system (VAS)	Yes [a]	Baseline and 2, 4 and 6 weeks	Difference of median combined symptom scores at baseline, 4 and 6 weeks between the mesalazine and placebo group.	Intensity of lower abdominal pain, frequency of lower abdominal pain, frequency of bloating/abdominal distension, flatulence, pressing during defaecation, sensation of incomplete evacuation after defaecation, nausea.	The combined symptom score at baseline was approximately 10% higher in the mesalazine than placebo group, but only the mean score for pressing during defecation was significantly higher in the mesalazine arm.<br>There was no statistically significant difference of median combined symptom scores between the two groups at 4 and 6 weeks.
Parente et al., 2013 (28)	Therapy Impact Questionnaire (TIQ), physical condition sub-score [b], using visual 1 to 10 points scales	No	Baseline and every 3 months until 24 months	Difference of the mean physical condition sub-scores at baseline and 24 months between the mesalazine and placebo group.	Faeces consistency, blood in faeces, abdominal/rectal pain and tenesmus, urgent evacuation level.	There was no statistically significant difference of the total physical condition sub-score between the two groups at baseline.<br>There was a significantly higher reduction of this sub-score in the mesalazine than placebo group at 24 months (p=0.022).

Study, year (reference)	Symptom assessment instrument	Validation of the instrument	Time of assessment	Method of Reporting	Symptoms reported	Results
Stollman et al., 2013 (29)	10-items global symptomatic score (GSS), using visual 0 to 6 points scales	No	Baseline, 10 days and 12, 26, 39 and 52 weeks	Median change of GSS between baseline and 12 weeks in the mesalazine and placebo group.<br>Difference of mean GSS at baseline, 12 and 52 weeks between the two groups.	Abdominal pain, abdominal tenderness, nausea/vomiting, bloating, pain/difficulty urinating, mucus in stool, constipation, diarrhea, urgency, painful straining.	In both groups, there was a statistically significant reduction of mean GSS at 12 weeks.<br>There was no statistically significant difference of mean GSS between the two groups at baseline, 12 and 52 weeks.
Tursi et al., 2013 (30)	Visual 0 to 10 points scales	No	Baseline and 1, 2, 6, 9 and 12 months	Difference in scores for each symptom at 12 months among the mesalazine, placebo and Lactobacillus casei subspecies DG group.	Abdominal pain, diarrhoea, constipation, rectal bleeding, bloating, sensation of incomplete evacuation, mucorrhoea	All participants were asymptomatic at baseline (score=0 for all symptoms).<br>There was no statistically significant difference in scores for each symptom among study groups at 12 months.
PREVENT1, 2014 (31)	Presence or absence of symptoms	No	Baseline and 14 study visits until 104 weeks	Difference of patients with abdominal symptoms at 104 weeks between the mesalazine and placebo group.	Abdominal symptoms.[c]	The percentage of participants with abdominal pain was similar between the mesalazine (11.7%) and placebo (10.9%) group at 104 weeks.
PREVENT2, 2014 (31)	Presence or absence of symptoms	No	Baseline and 14 study visits until 104 weeks	Difference of patients with abdominal symptoms at 104 weeks between the mesalazine and placebo group.	Abdominal symptoms. [c]	The percentage of participants with abdominal pain was similar between the mesalazine (11.9%) and placebo (8.5%) group at 104 weeks.
SAG-37, 2017 (32)	Patient Health Questionnaire 15 (PHQ-15), using visual 0 to 2 point scales	Yes [d]	Baseline and 12 months	Mean (SD) change in the PHQ-15 total score from baseline to 12 months	Stomach pain, back pain, pain in arms/legs/joints, menstrual cramps or other problems with periods (women only), headaches, chest pain, dizziness, fainting spells, feeling heart pound/race, shortness of breath, pain/problems during sexual intercourse, constipation/loose bowels/diarrhea, nausea/gas/indigestion, feeling tired/having low energy, trouble sleeping	There was no statistically significant difference between the mesalazine and placebo groups in mean change in total symptom score.
SAG-51, 2017 (32)	Patient Health Questionnaire 15 (PHQ-15), using visual 0 to 2 point scales	Yes [d]	Baseline and 12 months	Mean (SD) change in the PHQ-15 total score from baseline to 12 months	Stomach pain, back pain, pain in arms/legs/joints, menstrual cramps or other problems with periods (women only), headaches, chest pain, dizziness, fainting spells, feeling heart pound/race, shortness of breath, pain/problems during sexual intercourse, constipation/loose bowels/diarrhea, nausea/gas/indigestion, feeling tired/having low energy, trouble sleeping	There was no statistically significant difference between the mesalazine and placebo groups in mean change in total symptom score.

GSS, global symptomatic score.
[a] Instrument validated to measure generic health outcomes in all therapeutic areas.
[b] 11-items questionnaire: the sum of items 1-4 defines the physical condition sub-score, while the sum of items 5-11 defines the quality-of-life sub-score.
[c] Symptoms not specified.
[d] Instrument validated to measure mental disorders, functional impairment, and recent psychosocial stressors in psychiatry/psychology.

Supplementary Table 5. Comparative effectiveness of mesalazine versus control interventions by type of control intervention, stratified by subtype of diverticular disease.

Outcome analyzed, type of control intervention (reference)	Studies reporting outcome, *n*	Patients enrolled, *n*	Risk Ratio (95% Confidence Interval) [a]	Heterogeneity		Test for subgroup differences, p value
				Chi-square	I^2, %	
Number of patients achieving remission [b]						
Symptomatic uncomplicated diverticular disease						-
Mesalazine versus Lactobacillus casei subspecies DG	-	-	-	-	-	
Mesalazine versus rifaximin	-	-	-	-	-	
Mesalazine versus placebo(27)	1	123	1.04 (0.81 to 1.34)	-	-	
Mesalazine versus no treatment	-	-	-	-	-	
Acute uncomplicated diverticulitis						-
Mesalazine versus Lactobacillus casei subspecies DG	-	-	-	-	-	
Mesalazine versus rifaximin	-	-	-	-	-	
Mesalazine versus placebo(29)	1	81	2.67 (1.05 to 6.79)	-	-	
Mesalazine versus no treatment	-	-	-	-	-	
Number of patients with recurrence of disease						
Symptomatic uncomplicated diverticular disease						0.22
Mesalazine versus Lactobacillus casei subspecies DG(23, 30) [c]	2	166	0.74 (0.32 to 1.72)	0.06	0	
Mesalazine versus rifaximin	-	-	-	-	-	
Mesalazine versus placebo(30) [c]	1	101	0.33 (0.13 to 0.86)	-	-	
Mesalazine versus no treatment	-	-	-	-	-	
Acute uncomplicated diverticulitis						0.001
Mesalazine versus Lactobacillus casei subspecies DG	-	-	-	-	-	
Mesalazine versus rifaximin	-	-	-	-	-	
Mesalazine versus placebo(28, 29, 31, 32)	6	2030	1.12 (0.87 to 1.44)	7.95	37	
Mesalazine versus no treatment(22)	1	166	0.32 (0.18 to 0.57)	-	-	
Number of patients developing acute diverticulitis in symptomatic uncomplicated diverticular disease						0.80
Mesalazine versus Lactobacillus casei subspecies DG(23, 30) [c]	2	166	0.49 (0.05 to 4.58)	0.11	0	
Mesalazine versus rifaximin(25)	1	268	0.34 (0.04 to 3.21)	-	-	
Mesalazine versus placebo(30) [c]	1	101	0.15 (0.01 to 2.48)	-	-	
Mesalazine versus no treatment	-	-	-	-	-	
Number of patients needing surgery						
Symptomatic uncomplicated diverticular disease						-
Mesalazine versus Lactobacillus casei subspecies DG(30) [c, d]	1	106	-	-	-	
Mesalazine versus rifaximin(25) [d]	1	268	-	-	-	
Mesalazine versus placebo(30) [c]	1	101	0.63 (0.03 to 14.94)	-	-	
Mesalazine versus no treatment	-	-	-	-	-	
Acute uncomplicated diverticulitis						-
Mesalazine versus Lactobacillus casei subspecies DG	-	-	-	-	-	
Mesalazine versus rifaximin	-	-	-	-	-	
Mesalazine versus placebo(29, 31)	3	1263	1.41 (0.51 to 3.90)	0.97	0	
Mesalazine versus no treatment	-	-	-	-	-	
Number of patients needing hospitalization						
Symptomatic uncomplicated diverticular disease						-
Mesalazine versus Lactobacillus casei subspecies DG(23)	1	60	0.33 (0.01 to 7.87)	-	-	
Mesalazine versus rifaximin	-	-	-	-	-	
Mesalazine versus placebo	-	-	-	-	-	
Mesalazine versus no treatment	-	-	-	-	-	
Acute uncomplicated diverticulitis	-	-	-	-	-	-

Outcome analyzed, type of control intervention (reference)	Studies reporting outcome, *n*	Patients enrolled, *n*	Risk Ratio (95% Confidence Interval) [a]	Heterogeneity		Test for subgroup differences, p value
				Chi-square	I^2, %	
Number of patients with any adverse events						
Symptomatic uncomplicated diverticular disease						0.40
Mesalazine versus Lactobacillus casei subspecies DG	-	-	-	-	-	
Mesalazine versus rifaximin(25)	1	268	0.61 (0.15 to 2.50)	-	-	
Mesalazine versus placebo(27)	1	123	1.20 (0.58 to 2.47)	-	-	
Mesalazine versus no treatment	-	-	-	-	-	
Acute uncomplicated diverticulitis						0.09
Mesalazine versus Lactobacillus casei subspecies DG	-	-	-	-	-	
Mesalazine versus rifaximin	-	-	-	-	-	
Mesalazine versus placebo(28, 29, 31, 32)	6	2030	1.03 (0.97 to 1.09)	5.57	10	
Mesalazine versus no treatment(22)	1	166	1.98 (0.94 to 4.19)	-	-	
All-cause mortality						
Symptomatic uncomplicated diverticular disease						-
Mesalazine versus Lactobacillus casei subspecies DG(23, 30) [c, d]	2	166	-	-	-	
Mesalazine versus rifaximin(25) [d]	1	268	-	-	-	
Mesalazine versus placebo(27, 30) [c, d]	2	224	-	-	-	
Mesalazine versus no treatment	-	-	-	-	-	
Acute uncomplicated diverticulitis						-
Mesalazine versus Lactobacillus casei subspecies DG	-	-	-	-	-	
Mesalazine versus rifaximin	-	-	-	-	-	
Mesalazine versus placebo(29, 31, 32) [d]	4	1346	-	-	-	
Mesalazine versus no treatment(22)	1	166	0.52 (0.05 to 5.68)	-	-	
Diverticular disease related mortality						
Symptomatic uncomplicated diverticular disease						-
Mesalazine versus Lactobacillus casei subspecies DG(23, 30) [c, d]	2	166	-	-	-	
Mesalazine versus rifaximin(25) [d]	1	268	-	-	-	
Mesalazine versus placebo(27, 30) [c, d]	2	224	-	-	-	
Mesalazine versus no treatment	-	-	-	-	-	
Acute uncomplicated diverticulitis						-
Mesalazine versus Lactobacillus casei subspecies DG	-	-	-	-	-	
Mesalazine versus rifaximin	-	-	-	-	-	
Mesalazine versus placebo(29, 31, 32) [d]	4	1346	-	-	-	
Mesalazine versus no treatment(22) [d]	1	166	-	-	-	

Outcome analyzed, type of control intervention (reference)	Studies reporting outcome, *n*	Patients enrolled, *n*	Mean Difference (95% Confidence Interval)	Heterogeneity		Test for subgroup differences, p value
				Chi-square	I^2, %	
Global symptoms score [e]						
Symptomatic uncomplicated diverticular disease						-
Mesalazine versus Lactobacillus casei subspecies DG	-	-	-	-	-	
Mesalazine versus rifaximin(24, 25)	2	326	-1.01 (-1.51 to -0.52) [g]	2.93	66	
Mesalazine versus placebo	-	-	-	-	-	
Mesalazine versus no treatment	-	-	-	-	-	
Acute uncomplicated diverticulitis						-
Mesalazine versus Lactobacillus casei subspecies DG	-	-	-	-	-	
Mesalazine versus rifaximin	-	-	-	-	-	
Mesalazine versus placebo(28, 29)	2	153	-0.56 (-0.88 to -0.24) [g]	0.36	0	
Mesalazine versus no treatment	-	-	-	-	-	

Outcome analyzed, type of control intervention (reference)	Studies reporting outcome, *n*	Patients enrolled, *n*	Mean Difference (95% Confidence Interval)	Heterogeneity		Test for subgroup differences, p value
				Chi-Square	I^2, %	
Time to recurrence (days) [f]						
Symptomatic uncomplicated diverticular disease	-	-	-	-	-	-
Acute uncomplicated diverticulitis						-
Mesalazine versus Lactobacillus casei subspecies DG	-	-	-	-	-	
Mesalazine versus rifaximin	-	-	-	-	-	
Mesalazine versus placebo(28, 29, 32)	3	91	-30.04 (-55.18 to -4.90)	2.36	15	
Mesalazine versus no treatment	-	-	-	-	-	

[a] values <1 or >1 indicate the direction of effect for mesalazine or control interventions, respectively.
[b] values >1 or <1 indicate the direction of effect for mesalazine or control interventions, respectively.
[c] the number of participants with recurrence and the total number in the mesalazine arm from one study (30) have been halved to avoid double-counting.
[d] no event was reported in included studies.
[e] values <0 or >0 indicate the direction of effect for mesalazine or control interventions, respectively.
[f] values >0 or <0 indicate the direction of effect for mesalazine or control interventions, respectively.
[g] standardized mean difference.

Supplementary Table 6. Comparative effectiveness of mesalazine versus control interventions by follow-up duration, stratified by subtype of diverticular disease.

Outcome analyzed, follow-up duration (reference)	Studies reporting outcome, *n*	Patients enrolled, *n*	Risk Ratio (95% Confidence Interval) [a]	Heterogeneity		Test for subgroup differences, p value
				Chi-square	I^2, %	
Number of patients achieving remission [b]						
Symptomatic uncomplicated diverticular disease						-
Less than 3 months(27)	1	123	1.04 (0.81 to 1.34)	-	-	
Between 3 and 6 months	-	-	-	-	-	
Between 6 and 12 months	-	-	-	-	-	
More than 12 months	-	-	-	-	-	
Acute uncomplicated diverticulitis						-
Less than 3 months	-	-	-	-	-	
Between 3 and 6 months	-	-	-	-	-	
Between 6 and 12 months(29)	1	81	2.67 (1.05 to 6.79)	-	-	
More than 12 months	-	-	-	-	-	
Number of patients with recurrence of disease						
Symptomatic uncomplicated diverticular disease						-
Less than 3 months	-	-	-	-	-	
Between 3 and 6 months	-	-	-	-	-	
Between 6 and 12 months(23, 30)	2	216	0.52 (0.28 to 0.97)	0.26	0	
More than 12 months	-	-	-	-	-	
Acute uncomplicated diverticulitis						0.10
Less than 3 months	-	-	-	-	-	
Between 3 and 6 months	-	-	-	-	-	
Between 6 and 12 months(29, 32)	2	426	1.34 (0.89 to 2.02)	1.00	0	
More than 12 months(22, 28, 31, 32)	5	1770	0.78 (0.47 to 1.29)	21.81	82	
Number of patients developing acute diverticulitis in symptomatic uncomplicated diverticular disease						-
Less than 3 months	-	-	-	-	-	
Between 3 and 6 months	-	-	-	-	-	
Between 6 and 12 months(23, 25, 30)	3	484	0.26 (0.06 to 1.20)	0.30	0	
More than 12 months	-	-	-	-	-	
Number of patients needing surgery						
Symptomatic uncomplicated diverticular disease						-
Less than 3 months	-	-	-	-	-	
Between 3 and 6 months	-	-	-	-	-	
Between 6 and 12 months(25, 30) [c]	2	424	0.68 (0.03 to 16.39)	-	-	
More than 12 months	-	-	-	-	-	
Acute uncomplicated diverticulitis						0.59
Less than 3 months	-	-	-	-	-	
Between 3 and 6 months	-	-	-	-	-	
Between 6 and 12 months(29)	1	81	1.02 (0.22 to 4.78)	-	-	
More than 12 months(31)	2	1182	1.81 (0.47 to 7.01)	0.65	0	
Number of patients needing hospitalization						
Symptomatic uncomplicated diverticular disease						-
Less than 3 months	-	-	-	-	-	
Between 3 and 6 months	-	-	-	-	-	
Between 6 and 12 months(23)	1	60	0.33 (0.01 to 7.87)	-	-	
More than 12 months	-	-	-	-	-	
Acute uncomplicated diverticulitis	-	-	-	-	-	-

Outcome analyzed, follow-up duration (reference)	Studies reporting outcome, *n*	Patients enrolled, *n*	Risk Ratio (95% Confidence Interval) [a]	Heterogeneity		Test for subgroup differences, p value
				Chi-square	I^2, %	
Number of patients with any adverse events						
Symptomatic uncomplicated diverticular disease						0.40
Less than 3 months(27)	1	123	1.20 (0.58 to 2.47)	-	-	
Between 3 and 6 months	-	-	-	-	-	
Between 6 and 12 months(25)	1	268	0.61 (0.15 to 2.50)	-	-	
More than 12 months	-	-	-	-	-	
Acute uncomplicated diverticulitis						0.58
Less than 3 months	-	-	-	-	-	
Between 3 and 6 months(29)	1	81	1.15 (0.70 to 1.87)	-	-	
Between 6 and 12 months	1	345	1.08 (0.98 to 1.20)	-	-	
More than 12 months(22, 28, 31, 32)	5	1770	1.01 (0.92 to 1.11)	6.85	42	
All-cause mortality						
Symptomatic uncomplicated diverticular disease						-
Less than 3 months(27) [d]	1	123	-	-	-	
Between 3 and 6 months	-	-	-	-	-	
Between 6 and 12 months(23, 25, 30) [d]	3	484	-	-	-	
More than 12 months	-	-	-	-	-	
Acute uncomplicated diverticulitis						-
Less than 3 months	-	-	-	-	-	
Between 3 and 6 months	-	-	-	-	-	
Between 6 and 12 months(29, 32) [d]	2	426	-	-	-	
More than 12 months(22, 31, 32) [e]	3	1086	0.52 (0.05 to 5.68)	-	-	
Diverticular disease related mortality						
Symptomatic uncomplicated diverticular disease						-
Less than 3 months(27) [d]	1	123	-	-	-	
Between 3 and 6 months	-	-	-	-	-	
Between 6 and 12 months(23, 25, 30) [d]	3	484	-	-	-	
More than 12 months	-	-	-	-	-	
Acute uncomplicated diverticulitis						-
Less than 3 months	-	-	-	-	-	
Between 3 and 6 months	-	-	-	-	-	
Between 6 and 12 months(29, 32) [d]	2	426	-	-	-	
More than 12 months(22, 31, 32) [d]	3	1086	-	-	-	
**Outcome analyzed, follow-up duration (reference)**	**Studies reporting outcome, *n***	**Patients enrolled, *n***	**Standardized Mean Difference (95% Confidence Interval) [f]**	**Heterogeneity**		**Test for subgroup differences, p value**
				**Chi-square**	**I^2, %**	
Global symptoms score						
Symptomatic uncomplicated diverticular disease						0.09
Less than 3 months	-	-	-	-	-	
Between 3 and 6 months(24)	1	58	-0.70 (-1.23 to -0.17)			
Between 6 and 12 months(25)	1	268	-1.22 (-1.48 to -0.96)	-	-	
More than 12 months	-	-	-	-	-	
Acute uncomplicated diverticulitis						0.55
Less than 3 months	-	-	-	-	-	
Between 3 and 6 months(29)	1	61	-0.44 (-0.95 to 0.07)	-	-	
Between 6 and 12 months	-	-	-	-	-	
More than 12 months(28)	1	92	-0.64 (-1.06 to -0.22)	-	-	

[a] values <1 or >1 indicate the direction of effect for mesalazine or control interventions, respectively.
[b] values >1 or <1 indicate the direction of effect for mesalazine or control interventions, respectively.
[c] no event was reported in one included study (25).
[d] no event was reported in included studies.
[e] no event was reported in two included studies: PREVENT1(31) and SAG-51(32).
[f] values <0 or >0 indicate the direction of effect for mesalazine or control interventions, respectively.

Supplementary Table 7. Comparative effectiveness of mesalazine versus control interventions by dose of mesalazine, stratified by subtype of diverticular disease.

Outcome analyzed, dose of mesalazine (reference)	Studies reporting outcome, *n*	Patients enrolled, *n*	Risk Ratio (95% Confidence Interval) [a]	Heterogeneity		Test for subgroup differences, p value
				Chi-square	I^2, %	
Number of patients achieving remission [b]						
Symptomatic uncomplicated diverticular disease						-
From 800 to 1600 mg	-	-	-	-	-	
More than 1600 up to 2400 mg	-	-	-	-	-	
More than 2400 mg(27)	1	123	1.04 (0.81 to 1.34)	-	-	
Acute uncomplicated diverticulitis						-
From 800 to 1600 mg	-	-	-	-	-	
More than 1600 up to 2400 mg(29)	1	81	2.67 (1.05 to 6.79)	-	-	
More than 2400 mg	-	-	-	-	-	
Number of patients with recurrence of disease						
Symptomatic uncomplicated diverticular disease						-
From 800 to 1600 mg(23, 30)	2	216	0.52 (0.28 to 0.97)	0.26	0	
More than 1600 up to 2400 mg	-	-	-	-	-	
More than 2400 mg	-	-	-	-	-	
Acute uncomplicated diverticulitis						0.22
From 800 to 1600 mg(22, 28, 31, 32) [c, d]	5	1084	0.71 (0.39 to 1.29)	15.64	74	
More than 1600 up to 2400 mg(29, 31) [c]	3	669	1.19 (0.83 to 1.71)	1.11	0	
More than 2400 mg(31, 32) [c, d]	4	1146	1.28 (0.95 to 1.72)	0.11	0	
Number of patients developing acute diverticulitis in symptomatic uncomplicated diverticular disease						-
From 800 to 1600 mg(23, 25, 30)	3	484	0.26 (0.06 to 1.20)	0.30	0	
More than 1600 up to 2400 mg	-	-	-	-	-	
More than 2400 mg	-	-	-	-	-	
Number of patients needing surgery						
Symptomatic uncomplicated diverticular disease						-
From 800 to 1600 mg(25, 30) [e]	2	424	0.68 (0.03 to 16.39)	-	-	
More than 1600 up to 2400 mg	-	-	-	-	-	
More than 2400 mg	-	-	-	-	-	
Acute uncomplicated diverticulitis						0.78
From 800 to 1600 mg(31) [c]	2	588	2.35 (0.29 to 18.86)	0	0	
More than 1600 up to 2400 mg(29, 31) [c]	3	669	1.00 (0.31 to 3.29)	0.21	0	
More than 2400 mg(31) [c]	2	596	1.19 (0.20 to 7.20)	0.07	0	
Number of patients needing hospitalization						
Symptomatic uncomplicated diverticular disease						-
From 800 to 1600 mg(23)	1	60	0.33 (0.01 to 7.87)	-	-	
More than 1600 up to 2400 mg	-	-	-	-	-	
More than 2400 mg	-	-	-	-	-	
Acute uncomplicated diverticulitis	-	-	-	-	-	-
Number of patients with any adverse events						
Symptomatic uncomplicated diverticular disease						0.40
From 800 to 1600 mg(25)	1	268	0.61 (0.15 to 2.50)	-	-	
More than 1600 up to 2400 mg	-	-	-	-	-	
More than 2400 mg(27)	1	123	1.20 (0.58 to 2.47)	-	-	
Acute uncomplicated diverticulitis						0.97
From 800 to 1600 mg(22, 28, 31) [c]	4	846	1.00 (0.82 to 1.21)	4.93	39	
More than 1600 up to 2400 mg(29, 31) [c]	3	669	1.03 (0.90 to 1.17)	0.41	0	
More than 2400 mg(31, 32) [c]	3	941	1.01 (0.89 to 1.15)	3.64	45	

Outcome analyzed, dose of mesalazine (reference)	Studies reporting outcome, *n*	Patients enrolled, *n*	Risk Ratio (95% Confidence Interval)[a]	Heterogeneity		Test for subgroup differences, p value
				Chi-square	I^2, %	
All-cause mortality						
Symptomatic uncomplicated diverticular disease						-
From 800 to 1600 mg(23, 25, 30)[f]	3	484	-	-	-	
More than 1600 up to 2400 mg	-	-	-	-	-	
More than 2400 mg(27)[f]	1	123	-	-	-	
Acute uncomplicated diverticulitis						-
From 800 to 1600 mg(22, 31, 32)[c, d]	3	698	0.52 (0.05 to 5.68)	-	-	
More than 1600 up to 2400 mg(29, 31)[c, f]	2	375	-	-	-	
More than 2400 mg(31, 32)[c, d, f]	3	850	-	-	-	
Diverticular disease related mortality						
Symptomatic uncomplicated diverticular disease						-
From 800 to 1600 mg(23, 25, 30)[f]	3	484	-	-	-	
More than 1600 up to 2400 mg	-	-	-	-	-	
More than 2400 mg(27)[f]	1	123	-	-	-	
Acute uncomplicated diverticulitis						-
From 800 to 1600 mg(22, 31, 32)[c, d, f]	3	698	-	-	-	
More than 1600 up to 2400 mg(29, 31)[c, f]	2	375	-	-	-	
More than 2400 mg(31, 32)[c, d, f]	3	850	-	-	-	

Outcome analyzed, dose of mesalazine (reference)	Studies reporting outcome, *n*	Patients enrolled, *n*	Mean Difference (95% Confidence Interval)	Heterogeneity		Test for subgroup differences, p value
				Chi-square	I^2, %	
Global symptoms score[g]						
Symptomatic uncomplicated diverticular disease						-
From 800 to 1600 mg(24, 25)	2	326	-1.01 (-1.51 to -0.52)[i]	-	-	
More than 1600 up to 2400 mg	-	-	-			
More than 2400 mg	-	-	-	-	-	
Acute uncomplicated diverticulitis						0.55
From 800 to 1600 mg(28)	1	92	-0.64 (-1.06 to -0.22)[i]	-	-	
More than 1600 up to 2400 mg(29)	1	61	-0.44 (-0.95 to 0.07)[i]	-	-	
More than 2400 mg	-	-	-	-	-	
Time to recurrence (days)[h]						
Symptomatic uncomplicated diverticular disease	-	-	-	-	-	-
Acute uncomplicated diverticulitis						0.23
From 800 to 1600 mg(28, 32)	2	55	-61.99 (-164.82 to 40.83)	1.22	18	
More than 1600 up to 2400 mg(29)	1	21	-32.50 (-46.42 to -18.58)	-	-	
More than 2400 mg(32)	2	88	8.55 (-40.14 to 57.24)	0.78	0	

[a] values <1 or >1 indicate the direction of effect for mesalazine or control interventions, respectively.
[b] values >1 or <1 indicate the direction of effect for mesalazine or control interventions, respectively.
[c] the number of participants with recurrence and the total number in the placebo arm from two studies, PREVENT1 and PREVENT2(31), have been divided by three to avoid triple-counting.
[d] the number of participants with recurrence and the total number in the placebo arm from one study, SAG-51(32), have been divided by two to avoid double-counting
[e] no event was reported in one included study (25).
[f] no event was reported in included studies.
[g] values <0 or >0 indicate the direction of effect for mesalazine or control interventions, respectively.
[h] values >0 or <0 indicate the direction of effect for mesalazine or control interventions, respectively.
[i] standardized mean difference.

Supplementary Table 8. Comparative effectiveness of mesalazine versus control interventions by mode of administration of mesalazine, stratified by subtype of diverticular disease.

Outcome analyzed, mode of administration of mesalazine (reference)	Studies reporting outcome, *n*	Patients enrolled, *n*	Risk Ratio (95% Confidence Interval) [a]	Heterogeneity		Test for subgroup differences, p value
				Chi-square	I^2, %	
Number of patients achieving remission [b]						
Symptomatic uncomplicated diverticular disease						-
Continuous(27)	1	123	1.04 (0.81 to 1.34)	-	-	
Cyclic	-	-	-	-	-	
Acute uncomplicated diverticulitis						-
Continuous(29)	1	81	2.67 (1.05 to 6.79)	-	-	
Cyclic	-	-	-	-	-	
Number of patients with recurrence of disease						
Symptomatic uncomplicated diverticular disease						0.61
Continuous(23)	1	60	0.67 (0.21 to 2.13)	-	-	
Cyclic(30)	1	156	0.46 (0.22 to 0.98)	-	-	
Acute uncomplicated diverticulitis						0.16
Continuous(22, 29, 31, 32)	6	2104	0.97 (0.64 to 1.45)	22.15	77	
Cyclic(28)	1	92	0.48 (0.20 to 1.16)	-	-	
Number of patients developing acute diverticulitis in symptomatic uncomplicated diverticular disease						0.86
Continuous(23)	1	60	0.33 (0.01 to 7.87)	-	-	
Cyclic(25, 30)	2	424	0.24 (0.04 to 1.39)	0.26	0	
Number of patients needing surgery						
Symptomatic uncomplicated diverticular disease						-
Continuous	-	-	-	-	-	
Cyclic(25, 30) [c]	2	424	0.68 (0.03 to 16.39)	-	-	
Acute uncomplicated diverticulitis						-
Continuous(29, 31)	3	1263	1.41 (0.51 to 3.90)	0.97	0	
Cyclic	-	-	-	-	-	
Number of patients needing hospitalization						
Symptomatic uncomplicated diverticular disease						-
Continuous(23)	1	60	0.33 (0.01 to 7.87)	-	-	
Cyclic	-	-	-	-	-	
Acute uncomplicated diverticulitis	-	-	-	-	-	-
Number of patients with any adverse events						
Symptomatic uncomplicated diverticular disease						0.40
Continuous(27)	1	123	1.20 (0.58 to 2.47)	-	-	
Cyclic(25)	1	268	0.61 (0.15 to 2.50)	-	-	
Acute uncomplicated diverticulitis						0.16
Continuous(22, 29, 31, 32)	6	2104	1.03 (0.95 to 1.13)	6.14	35	
Cyclic(28)	1	92	0.73 (0.45 to 1.19)	-	-	
All-cause mortality						
Symptomatic uncomplicated diverticular disease						-
Continuous(23, 27) [d]	2	183	-	-	-	
Cyclic(25, 30) [d]	2	424	-	-	-	
Acute uncomplicated diverticulitis						-
Continuous(22, 29, 31, 32) [e]	5	1512	0.52 (0.05 to 5.68)	-	-	
Cyclic	-	-	-	-	-	

Outcome analyzed, mode of administration of mesalazine (reference)	Studies reporting outcome, *n*	Patients enrolled, *n*	Risk Ratio (95% Confidence Interval) [a]	Heterogeneity		Test for subgroup differences, p value
				Chi-square	I^2, %	
Diverticular disease related mortality						
Symptomatic uncomplicated diverticular disease						-
Continuous(23, 27) [d]	2	183	-	-	-	
Cyclic(25, 30) [d]	2	424	-	-	-	
Acute uncomplicated diverticulitis						-
Continuous(22, 29, 31, 32) [d]	5	1512	-	-	-	
Cyclic	-	-	-	-	-	

Outcome analyzed, mode of administration of mesalazine (reference)	Studies reporting outcome, *n*	Patients enrolled, *n*	Mean Difference (95% Confidence Interval)	Heterogeneity		Test for subgroup differences, p value
				Chi-square	I^2, %	
Global symptoms score [f]						
Symptomatic uncomplicated diverticular disease						-
Continuous	-	-	-	-	-	
Cyclic(24, 25)	2	326	-1.01 (-1.51 to -0.52) [h]	-	-	
Acute uncomplicated diverticulitis						0.55
Continuous(29)	1	61	-0.44 (-0.95 to 0.07) [h]	-	-	
Cyclic(28)	1	92	-0.64 (-1.06 to -0.22) [h]	-	-	
Time to recurrence (days) [g]						
Symptomatic uncomplicated diverticular disease	-	-	-	-	-	-
Acute uncomplicated diverticulitis						0.22
Continuous(29, 32)	2	72	-30.96 (-44.49 to -17.44)	0.84	0	
Cyclic(28)	1	19	-150.80 (-340.42 to 38.82)	-	-	

[a] values <1 or >1 indicate the direction of effect for mesalazine or control interventions, respectively.
[b] values >1 or <1 indicate the direction of effect for mesalazine or control interventions, respectively.
[c] no event was reported in one included study (25).
[d] no event was reported in included studies.
[e] no event was reported in four included studies: : Stollman et al.(29), PREVENT1(31), SAG-37(32) and SAG-51(32).
[f] values <0 or >0 indicate the direction of effect for mesalazine or control interventions, respectively.
[g] values >0 or <0 indicate the direction of effect for mesalazine or control interventions, respectively.
[h] standardized mean difference.

Supplementary Figure 1. a. Risk of bias graph: review authors' judgements about each risk of bias item presented as percentages across all included studies; b. Risk of bias summary: review authors' judgements about each risk of bias item for each included study.

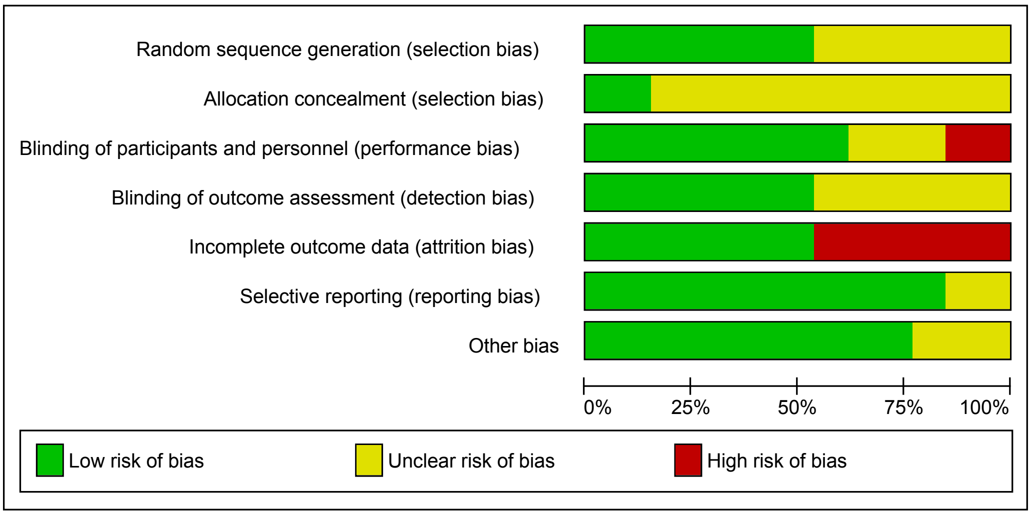

a

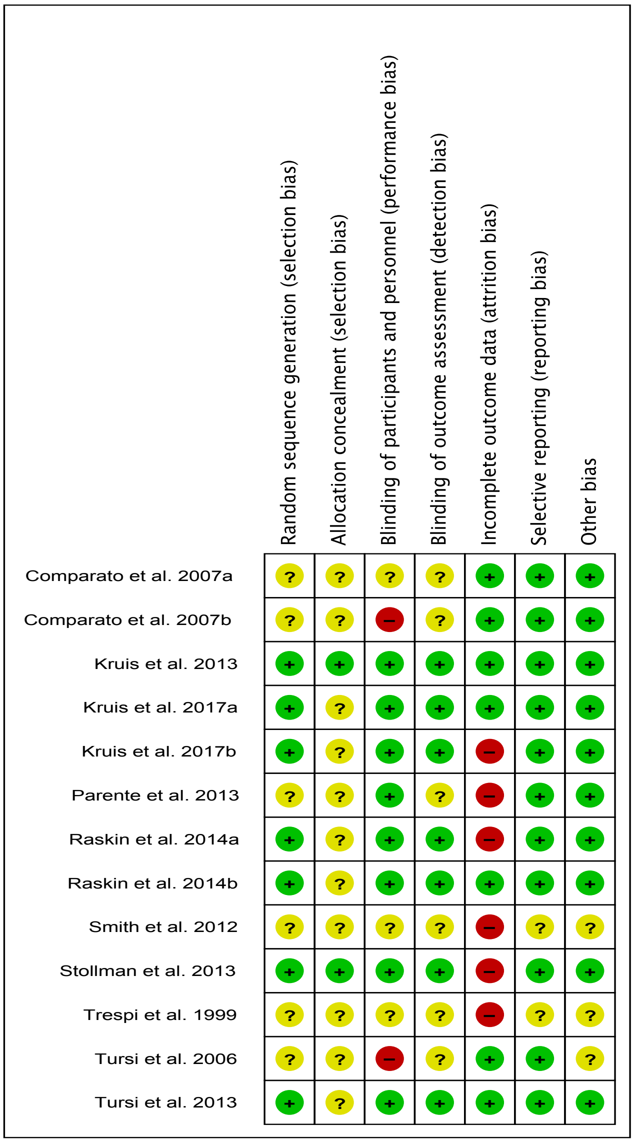

b
